# Combatting pulmonary fibrosis with *Astragalus membranaceus*: A review of active components and multifaceted mechanisms

**DOI:** 10.1016/j.chmed.2026.04.007

**Published:** 2026-05-01

**Authors:** Xiujuan Sun, Jingwen Zhao, Yiteng Ma, Zhihao Kong, Guangyue Su

**Affiliations:** a Shenyang Pharmaceutical University, Shenyang 110016, China; b State Key Laboratory of Druggability Evaluation and Systematic Translational Medicine (Tianjin Institute of Pharmaceutical Research), Tianjin 300301, China; c Key Laboratory of Innovative Traditional Chinese Medicine for Major Chronic Diseases of Liaoning Province, Shenyang 110016, China

**Keywords:** anti-fibrotic mechanisms, *Astragalus* active ingredients, *Astragalusmembranaceus* (Fisch.) Bunge, multi-targeted effects, pulmonary fibrosis

## Abstract

Pulmonary fibrosis (PF) is a chronic progressive lung disease characterized by excessive deposition of extracellular matrix (ECM) and loss of lung function. Treatment options are limited and can only delay disease progression, making the development of novel therapeutic strategies crucial. *Astragalus membranaceus* (AM), a traditional Chinese medicinal herb renowned for its lung-tonifying and immunomodulatory properties, contains bioactive constituents such as flavonoids, saponins, and polysaccharides. These components exhibit anti-inflammatory, antioxidant, anti-fibrotic, and immunoregulatory activities, demonstrating therapeutic potential in PF. This review systematically elucidates the complex pathogenesis of PF, comprehensively summarizes the principal chemical constituents of AM, and highlights its multi-targeted anti-fibrotic mechanisms of action. Research demonstrates that AM and its bioactive constituents exert anti-fibrotic effects through multiple pharmacological actions: inhibiting fibroblast activation, reversing epithelial-mesenchymal transition (EMT), modulating immune-inflammatory responses, protecting alveolar epithelial cells (AECs), and ameliorating mitochondrial function. Through the synergistic interaction of its multiple components and multi-targeted effects, AM demonstrates significant advantages in combating PF. This review provides a comprehensive theoretical basis for deepening the understanding of AM anti-fibrotic mechanisms and offers practical guidance for developing novel anti-fibrotic agents based on traditional Chinese medicine and its bioactive constituents.

## 1 Introduction

Pulmonary fibrosis (PF) is a chronic, progressive lung disease characterized pathologically by the activation and proliferation of fibroblasts and myofibroblasts, excessive deposition of extracellular matrix (ECM), and aggravated vascular remodeling. These pathological changes induce a progressive decline in lung function, ultimately leading to respiratory failure and even life-threatening outcomes. PF carries an extremely poor prognosis, with a median survival of only 3–5 years following diagnosis (Cheng et al., 2024). Despite significant advances has been made in understanding PF pathogenesis, there remains a paucity of effective clinical treatments to achieve a curative effect. Pirfenidone and nintedanib are two pharmaceutical agents approved by the United States Food and Drug Administration (FDA) for the treatment of idiopathic PF. However, both agents have prominent adverse effects, are costly, and fail to reverse disease progression or improve patient survival rates (Provenzani et al., 2025). Lung transplantation is currently an effective treatment for end-stage PF. However, due to donor shortages, exorbitant medical costs, age-related eligibility criteria for patients, and the risk of immune rejection, only a small subset of patients can undergo this treatment. Accordingly, the primary focus of PF clinical management is to alleviate patient symptoms and slow fibrosis progression.

It is evident that traditional Chinese medicine (TCM) and its active components exhibit distinctive advantages in the prevention and treatment of complex diseases, attributable to their multi-component and multi-target intervention characteristics. In TCM theory, PF is often classified as ‘*fei wei*’ (lung-defense) or ‘*fei bi*’ (lung impediment), with its core pathological mechanism summarized as ‘deficiency in the root with excess in the manifestation’. The root deficiency primarily affects the lungs and kidneys, while the excess in manifestation is characterized by pathological factors such as phlegm-dampness and blood stasis. This pathological mechanism aligns with the findings of modern medicine, which has revealed repeated alveolar epithelial injury-abnormal repair, impaired alveolar epithelial barrier function, reduced regenerative and repair capacity, mitochondrial dysfunction, persistent inflammatory storms, excessive ECM deposition, and epithelial-mesenchymal transition (EMT) and other pathological processes.

*Astragalus membranaceus* (Fisch.) Bunge (AM), a member of the leguminous plant family, has a long history of medicinal use in China. The *Shennong’s Classic of Material Medica* is an ancient Chinese medical monograph, records that AM treats chronic sores and abscesses, promotes pus discharge, and relieves pain. Similarly, the *Compendium of Materia Medica* regards it as the premier tonic herb, emphasizing its core effects of benefiting *qi*, consolidating the exterior, detoxifying, and promoting tissue regeneration. In clinical practice, AM is frequently employed to address deficiencies of *qi* and blood, and its application extends to the treatment of tumors, inflammatory diseases, and viral and bacterial infections (Sheng et al., 2024, Zhang et al., 2025b). Although numerous studies from multiple perspectives have confirmed the anti-PF potential of AM and its active components, the existing research findings are scattered and lack systematic review and integration. This review aims to integrate the extant evidence in order to provide a theoretical basis and clear research and development pathway for the modern scientific interpretation of AM in anti-PF therapy and the development of innovative drugs.

## 2 Pathogenesis of PF

The hallmark features of PF encompass excessive deposition of the ECM, destruction of normal lung parenchymal structure, and progressive loss of lung function. A number of factors have been identified as contributing to the development and progression of PF. The progression of PF can be promoted by a number of factors, including long-term inhalation of silica dust, tobacco smoke, viral infections, aging, and genetic susceptibility (Kishore & Petrek, 2021). The pathogenesis of PF remains to be fully elucidated, but it is currently widely believed to result from the combined effects of repeated micro-injuries to alveolar epithelial cells (AECs), the release of inflammatory mediators, activation of pro-fibrotic cytokines, and EMT.

### 2.1 EMT

EMT is the initiating event in fibrosis, typically characterized by the upregulation of mesenchymal markers and the downregulation of epithelial markers (Zhang, Jiao, Wu, & Sun, 2019). Research suggests that EMT plays an important role in the development of PF and is closely associated with multiple signaling pathways, including extracellular signal-regulated kinase (ERK), neurogenic locus notch homolog protein (Notch), and nuclear factor-*κ*B (NF-*κ*B). These pathways can activate EMT-related transcription factors, which, upon binding to the promoter of epithelial cadherin (E-cadherin), disrupt intercellular adhesion and degrade E-cadherin (Jeong, Kim, Park, & Chung, 2019).

#### 2.1.1 Transforming growth factor-β (TGF-β)

TGF-*β*, a pivotal regulator of cell differentiation, migration, proliferation, and gene expression, has been identified as a key mediator in the development of PF (Yin et al., 2024). It has been demonstrated that this process can induce EMT of terminally differentiated AECs through both the classical SMAD family member (Smad)-dependent pathway and the non-classical non-Smad pathway. Furthermore, TGF-*β* has been demonstrated to induce fibroblast-to-myofibroblast transition (FMT) via these two pathways. This process activates myofibroblasts to synthesize excessively ECM, while concurrently inhibiting ECM degradation. The resultant effect of this process is lung injury and PF (Fattet & Yang, 2020).

Fibroblasts, the primary cells responsible for synthesizing the ECM, function as the core effector cells in the process of fibrosis. Furthermore, they are the primary targets of TGF-*β* action under fibrotic conditions. Abnormal regulation of the TGF-*β*1/Smad pathway is an important mechanism in the development of tissue fibrosis. During the activation of myofibroblasts, TGF-*β*1 has been observed to directly induce the transcriptional expression of pro-fibrotic factors through this pathway. These factors play a pivotal role in the progression of PF by regulating EMT, AECs dysfunction, myofibroblast differentiation, and cellular senescence (Kadota et al., 2021). It is currently hypothesized that the primary sources of TGF-*β* are alveolar epithelial type II (ATII) cells and macrophages. Upon binding to its receptors, TGF-*β* has been observed to promote the expression of pro-fibrotic factors, including Smad2, Smad3, and connective tissue growth factor (CTGF). Furthermore, cross-regulation between TGF-*β* and the wingless-type mouse mammary tumor virus integration site family/beta-catenin (Wnt/*β*-catenin) signaling pathway has been demonstrated to impact epithelial cell function. The present study investigates the targeting of the TGF-*β*-mediated fibrotic pathway by AM and its active components (Ren et al., 2020). It demonstrates the ability of these substances to modulate the expression of related signaling molecules and to block TGF-*β*-induced EMT and fibroblast activation. This latter process is one of the important targets of the anti-PF action of AM.

#### 2.1.2 Hedgehog signaling pathway and Notch signaling pathway

A plethora of studies have demonstrated that both the Hedgehog and Notch signaling pathways are pivotal in the development of PF, particularly in the context of regulating EMT and fibroblast activation. In patients diagnosed with PF and the bleomycin (BLM)-induced mouse model of PF, significant activation of the Hedgehog signaling pathway has been demonstrated. This pathway has been demonstrated to facilitate the transformation of fibroblasts into myofibroblasts, thereby promoting ECM deposition. Inhibition of this pathway has been shown to retard the progression of PF (Chen et al., 2018).

Further studies have found that elevated levels of TGF-*β* can activate the Hedgehog signaling pathway by promoting the nuclear translocation and transcriptional activation of type I collagen in lipopolysaccharide (LPS)-induced mouse models. This process is crucial for the pathological changes mediated by this pathway (Zhang et al., 2022). Concurrently, elevated expression of the Notch signaling pathway has been observed in rat models of PF. This pathway has been demonstrated to directly induce the transformation of fibroblasts into myofibroblasts, promote TGF-*β* activation, and Smad3 phosphorylation. In addition, it has been shown to regulate the EMT process. Furthermore, the Notch signaling pathway has been demonstrated to indirectly promote the occurrence and progression of EMT by enhancing the TGF-*β*/Smad signaling pathway and through crosstalk with other signaling pathways, such as NF-*κ*B and Wnt/*β*-catenin (Vera et al., 2021; Xiao et al., 2023a).

In the progression of PF, the reactivation of the Hedgehog and Notch signaling pathways signifies that the repair process of lung tissue after injury deviates from the normal trajectory, shifting towards an abnormal ‘developmental-type’ repair pathway. These two pathways intertwine with the TGF-*β* signaling pathway, forming a complex regulatory network that collectively mediates alveolar epithelial cell dysfunction and excessive activation of stromal cells. It is hypothesized that AM and its active components can intervene in the activation states of these intersecting signaling pathways, disrupting the pro-fibrotic regulatory network they mediate, thereby slowing the progression of PF.

### 2.2 Damaged alveolar epithelial cells (AECs)

The dysfunction of AECs is recognized as a critical driver of PF pathogenesis. The ATI and ATII cells, and preservation of this epithelial barrier integrity is essential for normal lung function (Shao et al., 2024).

In the early stages of injury, damaged AECs lose their barrier function, enabling pathogens and pro-inflammatory factors to invade the lung tissue. These invading substances then trigger an inflammatory response, activating fibroblasts and immune cells and accelerating the process of fibrotic remodeling. The interaction between AECs and immune cells (such as alveolar macrophages, neutrophils and T lymphocytes) is central to the regulatory network that governs the repair mechanism following injury (Parimon et al., 2023).

When the alveolar epithelium is damaged, ATII cells are activated as alveolar stem cells and transdifferentiate into ATI cells, thereby reconstructing the alveolar epithelial barrier. The damaged epithelial cells release chemokines, such as C-C motif chemokine ligand 2 (CCL2) and C-X-C motif chemokine ligand 12 (CXCL12), which recruit immune cells, including monocytes/macrophages and neutrophils, to the injury site. These immune cells promote tissue repair by clearing cellular debris and secreting anti-inflammatory factors, such as interleukin-10 (IL-10) and transforming growth factor-beta 3(TGF-*β*3). As the repair process progresses, macrophages polarize from the pro-inflammatory M1 type to the anti-inflammatory or reparative M2 type. They secrete matrix metalloproteinases (MMPs) to degrade the ECM at the injury site, creating conditions for normal tissue reconstruction. Additionally, epithelial cells and fibroblasts maintain a dynamic balance of ECM synthesis and degradation by secreting growth factors, ultimately restoring the normal structure and function of lung tissue (Blokland et al., 2020).

In the pathological state of PF, this damage-repair cycle becomes imbalanced, evolving into a vicious cycle of ‘repeated injury-abnormal repair’, ultimately leading to excessive ECM deposition and lung structural damage (Lv et al., 2022). Macrophage polarization is disrupted, resulting in the sustained M1 pro-inflammatory state and the secretion of pro-fibrotic factors, thereby creating a persistent inflammatory-fibrotic microenvironment (Meli et al., 2020, Rui et al., 2022). Excessive production of pro-fibrotic factors, such as TGF-*β*1, has been demonstrated to cause fibroblasts to abnormally activate into myofibroblasts and proliferate extensively, leading to continuous ECM deposition. The excessive deposition of ECM has been demonstrated to result in the thickening of the basement membrane, a process that has been shown to directly impede alveolar epithelial function and to further activate epithelial cells, fibroblasts, and signaling pathways such as hypoxia-inducible factor-1 alpha (HIF-1*α*). This alteration of tissue mechanical properties, in turn, has been demonstrated to cause local hypoxia, a process that has been shown to further exacerbate the release of pro-fibrotic factors and abnormal angiogenesis (Cheng et al., 2021), thus forming a vicious cycle of ‘injury-inflammation-fibrosis-reinjury’.

Damaged AECs and dysregulated immune cells in the senescence-associated secretory phenotype (SASP) continuously secrete pro-inflammatory and pro-fibrotic factors, ultimately leading to excessive remodeling and structural disorganization of lung tissue, driving disease progression (Oh et al., 2020).

In accordance with the principles of TCM, the recurrence of damage and impaired regeneration of AECs are characteristic manifestations of lung *qi* deficiency. The weakened *qi* is incapable of driving recovery, which in turn leads to abnormal repair of AECs and progression of EMT (Sun et al., 2016). It is posited that the active components of AM, in accordance with the principle of “tonifying *qi* and consolidating the exterior”, are instrumental in enhancing the self-repair capacity of damaged AECs. Moreover, it is further postulated that, based on the principle of “supporting detoxification and removing stasis”, these components intervene in the EMT process, thereby disrupting the vicious cycle of “*qi* deficiency leading to stasis, and stasis causing pathological changes” by regulating related signaling pathways. This, in turn, is believed to exert anti-fibrotic effects from the root.

### 2.3 Inflammation response

The *Compendium of Materia Medica* is an extensive compendium of medicinal substances, with records of their traditional uses, properties and efficacy. It records the traditional use of AM in the treatment of detoxification and the promotion of pus discharge. This suggests that AM may have a potential role in the clearance of “heat toxins” from the lungs. Contemporary research has demonstrated that its active components are capable of inhibiting key inflammatory pathways such as the NLR family pyrin domain containing 3 (NLRP3) inflammasome and the NF-*κ*B pathways through multiple targets, and downregulating characteristic cytokines of “heat toxins” such as tumor necrosis factor-*α* (TNF-*α*), IL-1*β*, and IL-6, thereby interrupting the inflammation-fibrosis transition pathway. This is consistent with the therapeutic strategy of clearing heat and detoxifying to protect the lung network (Ke et al., 2023, Qin et al., 2023). The inflammatory response is a pivotal mechanism by which the body eliminates pathogens and other inflammation-triggering factors, and it plays a significant role in the repair of tissue and organ damage. The inflammatory response is a multi-step process that is regulated by various mediators. Damage to lung tissue, whether caused by exogenous or endogenous stimuli, instigates an inflammatory response throughout the body. This response can further contribute to the progression of PF. In a state of lung *qi* deficiency, the lung’s protective barriers are impaired, making pulmonary epithelial cells more vulnerable to fibrogenic factors. Persistent inflammation exacerbates PF progression, as inflammatory cell infiltration and cytokine release induce irreversible lung structural and functional damage. Notably, the inflammatory response in PF is regulated by multiple signaling pathways, among which the Wnt/*β*-catenin pathway is a key regulatory component that participates in the progression of inflammatory responses and fibrosis.

#### 2.3.1 Wnt/β-catenin pathway

The Wnt/*β*-catenin signaling pathway has been demonstrated to play a pivotal role in the regulation of diverse physiological processes, including embryonic development, organ formation, and cell apoptosis. However, in pathological conditions such as inflammatory diseases, neurological disorders, metabolic diseases, and tumors, the regulation of this pathway can become abnormal. During the development of PF, the Wnt/*β*-catenin signaling pathway has been observed to promote the transformation of fibroblasts into myofibroblasts, induce EMT, and enhance collagen synthesis and ECM deposition. As demonstrated by the extant research, the inhibition of the Wnt/*β*-catenin signaling pathway has been shown to be an effective means of preventing the progression of PF (Sun et al., 2020). This pathway has been demonstrated to promote inflammatory responses by transactivating NF-*κ*B and upregulating cyclooxygenase (COX) expression, while also increasing the phosphorylation levels of protein kinase B (Akt) and ERK. In AECs, the activation of the Wnt/*β*-catenin signaling pathway has been observed to promote the expression of IL-1*β*, thereby exacerbating the inflammatory response (Li et al., 2021). It has been demonstrated that AM and its active components can achieve synergistic anti-inflammatory and anti-fibrotic effects by inhibiting the abnormal activation of the Wnt/*β*-catenin signaling pathway and regulating the release of its downstream inflammatory factors.

#### 2.3.2 Interleukin

It has been demonstrated through a range of studies that in mouse models of fibrosis induced by BLM and LPS, reactive oxygen species (ROS) have been found to activate the NLRP3 inflammasome. The inflammasome has been identified as a contributing factor to the pathogenesis of PF through the IL-1*β*/IL-1 receptors (IL-1Rs)/myeloid differentiation factor 88 (MyD88)/NF-*κ*B signaling pathway (Song et al., 2016).

IL-1*β* is a pleiotropic inflammatory factor that plays an important role in the progression of PF and can dose-dependently stimulate collagen expression *in vitro* experiments. The production of IL-1*β* is typically the result of the synergistic action of two signaling pathways. The first pathway depends on a multiprotein complex that includes cysteine-aspartic protease-1 (Caspase-1), also known as the inflammasome. This complex generates biologically active IL-1*β* by cleaving the IL-1*β* precursor protein. The second pathway regulates IL-1*β* expression through a NF-*κ*B-dependent signaling pathway (Chen et al., 2021). Excessive release of IL-1*β* has been demonstrated to trigger inflammatory responses, inflammatory cell death, and diffuse organ damage. IL-6 is a multifunctional cytokine that plays a crucial role in regulating various cellular processes, including cell survival, differentiation, and proliferation. It is a potent pro-fibrotic and pro-inflammatory cytokine that is closely associated with the pathogenesis of various conditions, including tumors, inflammatory diseases and neurological disorders. Signal transducer and activator of transcription 3 (STAT3) is a member of the transcription factor family, playing a crucial role in cellular aging and participating in the development of multiple diseases. A plethora of studies have indicated that phosphorylated STAT3 levels are considerably elevated in the fibroblasts of patients diagnosed with PF. Moreover, activated STAT3 has been shown to further promote the progression of fibrosis. The activation of STAT3 is contingent upon the involvement of IL-6 family cytokines. In BLM-induced PF mouse models, IL-6 can activate the STAT3 signaling pathway subsequent to binding to the receptor glycoprotein 130 (gp130) (Han et al., 2023). IL-6 has been demonstrated to transmit local inflammatory signals to the whole body, thereby recruiting immune cells. These recruited immune cells secrete more inflammatory factors, triggering an inflammatory cascade that ultimately drives the progression of fibrosis. It has been demonstrated by other studies that IL-4, IL-24, IL-13 and IL-19 have the capacity to promote the progression of PF, whereas IL-33 exerts an inhibitory effect on the process of PF (Rao et al., 2021, Stephenson et al., 2023). As demonstrated in the relevant literature, the active components of AM have the capacity to inhibit the inflammatory cascade by targeting and regulating the expression of pro-inflammatory interleukins and related signaling pathways, thereby blocking the transition from inflammation to fibrosis.

#### 2.3.3 TNF-α

TNF-*α* is a multifunctional pro-inflammatory cytokine that plays a pivotal role in the development of PF. The production of this substance is primarily initiated by macrophages, which are stimulated by LPS. This factor has been demonstrated to act as an activator of phagocytosis and to regulate the immune response of the body during infection (Zhang, Wang, & Zhao, 2020). The extant literature suggests that TNF-*α* is primarily secreted locally by fibroblasts. However, some studies also indicate that, in the pathogenesis of PF, TNF-*α* mainly originates from radioresistant non-hematopoietic cells. The expression of TNF-*α* has been demonstrated to trigger acute inflammatory responses following tissue injury, as well as continuously releasing chronic inflammatory signals. When the intensity of these signals exceeds the threshold that controls the fibrotic response, it can lead to uncontrolled tissue repair and ultimately result in fibrosis. TNF-*α* exists in two forms: transmembrane TNF-*α* (tmTNF-*α*) and soluble TNF-*α* (sTNF-*α*). TNF-*α* exerts its effects by binding to its receptors, tumor necrosis factor receptor 1 (TNFR1) and TNFR2. Binding to TNFR1 has been shown to induce cell death and inflammatory responses, while tmTNF-*α* can trigger inflammation, though it is insufficient to mediate the transition to fibrosis. In contrast, sTNF-*α* has been demonstrated to promote lymphocyte recruitment, subsequently induce the expression of TGF-*β*1, and promote the formation of fibrotic lesions (Maranatha, Hasan, & Bakhtiar, 2022).

**Fig. 1:**
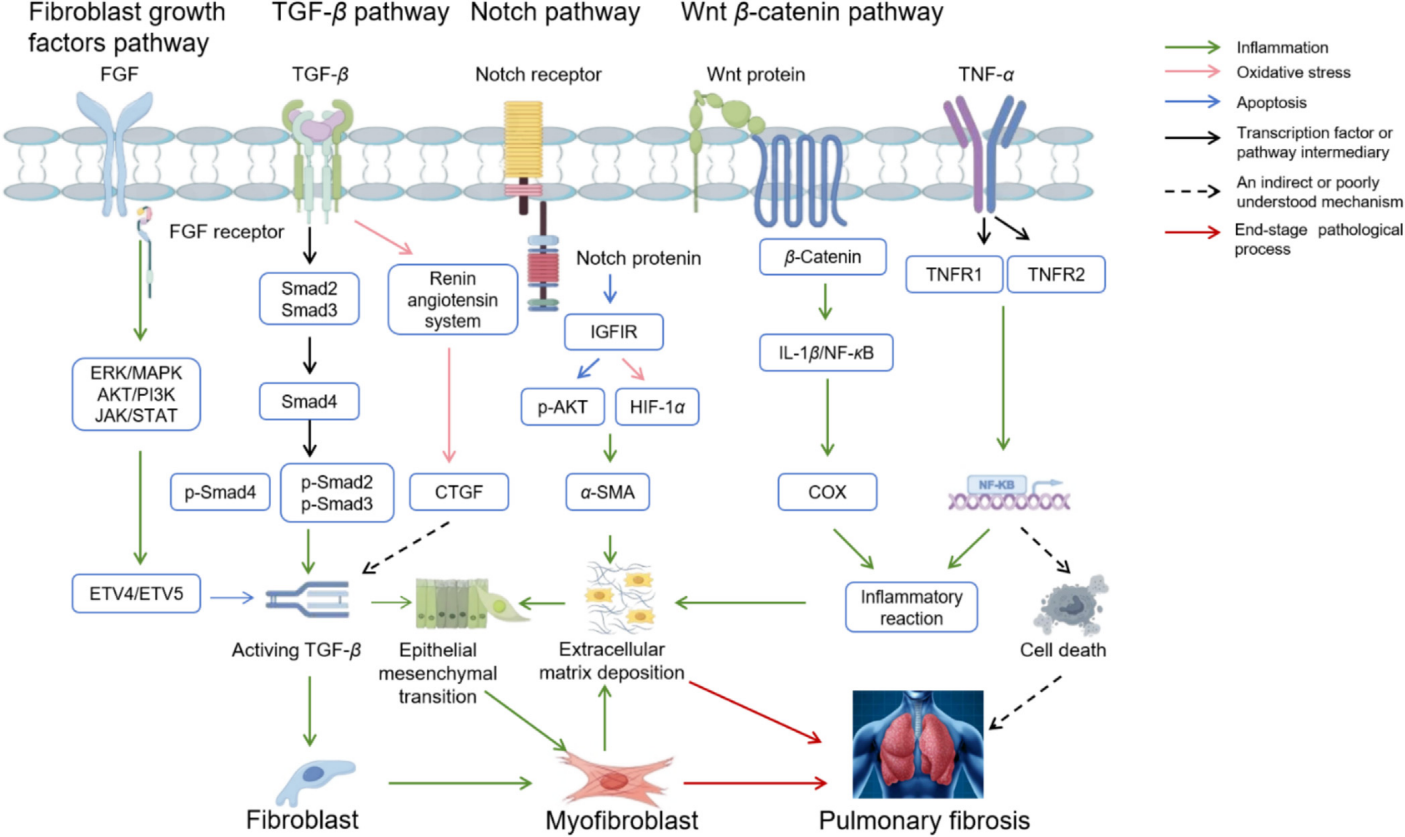
Signaling pathways associated with PF. MAPK: mitogen-activated protein kinase; PI3K: phosphoinositide 3-kinase; JAK: Janus kinase; ETV: ETS variant transcription factor; IGFIR: insulin-like growth factor 1; *α*-SMA: alpha-smooth muscle actin; CTGF: connective tissue growth factor.

It illustrates the core signaling network regulating PF (Fig. 1). The fibroblast growth factor (FGF) pathway drives early EMT and myofibroblast activation. As a central cascade, the TGF-*β* pathway promotes EMT and ECM deposition via the Smad axis and oxidative stress. The Notch, Wnt/*β*-catenin, and TNF-*α* pathways respectively modulate myofibroblast activation, trigger inflammation, and induce cell death, collectively exacerbating fibrosis. Crosstalk among these pathways through inflammation and oxidative stress synergistically drives EMT, myofibroblast activation, and ECM accumulation, providing a mechanistic framework for understanding PF pathogenesis.

### 2.4 Mitochondrial dysfunction

Mitochondrial dysfunction has emerged as a pivotal factor in the etiology of various diseases, including cancer, neurodegenerative diseases, cardiovascular diseases, and lung diseases (Zhong et al., 2024). Energy metabolism represents one of the core functions of mitochondria, and mitochondrial dysfunction is recognized as a hallmark of cellular aging. The mitochondrial respiratory chain plays a pivotal role in maintaining intracellular redox balance and signal transduction. It is also the primary source of ROS within cells. It is evident that persistent oxidative stress has the capacity to induce senescence or apoptosis in AECs. Furthermore, it has been demonstrated that this can result in the activation of multiple pro-fibrotic signaling pathways, including TGF-*β* and the NLRP3 inflammasome. Consequently, this creates a pro-inflammatory and pro-fibrotic microenvironment.

It is evident that a considerable metabolic reprogramming phenomenon occurs during the progression of PF. Dysfunctional AECs and activated fibroblasts shift their energy metabolism from efficient mitochondrial oxidative phosphorylation to less efficient glycolysis (Summer et al., 2024, Zhao et al., 2024). It has been demonstrated that AM and its active components can alleviate the formation of a pro-fibrotic microenvironment induced by oxidative stress by improving mitochondrial function and scavenging excessive ROS. This is one of the important anti-PF mechanisms of AM. Mitochondria, as the cell’s energy factories, when dysfunctional, lead to an energy crisis and oxidative stress, which are closely related to the pathogenesis described in TCM as “*qi* and *yin* deficiency” and “virtual fire generating internally” (Glancy, 2020). The effects of AM in benefiting *qi* and nourishing *yin* may improve mitochondrial biogenesis and function, enhance antioxidant defenses, alleviate *qi* deficiency, provide an energy basis for cell repair, and eliminate “virtual fire”, thereby exerting anti-fibrotic effects.

## 3 Active constituents of AM

Over 400 chemical components have been isolated and identified from AM, including polysaccharides, saponins, flavonoids, alkaloids, amino acids, and other metabolites. Tables S1–S3 present a comprehensive list of the various components isolated from AM, including flavonoids (F1−F79), saponins (S1−S73), and polysaccharides (A1−A20). The chemical structures of the flavonoids and saponins found in the AM plant are presented in Tables S4–S5.

### 3.1 *Astragalus* flavonoids

Total flavonoids of *Astragalus* (TFA) represent a major class of bioactive components in AM. The isolated flavonoid compounds include isoflavones (e.g., calycosin and formononetin), isoflavone glycosides (e.g., calycosin-7-*O*-*β*-*D*-glucoside and formononetin-7-*O*-*β*-*D*-glucoside), and flavonols (e.g., formononetin-7-hydroxy-4′-methoxyisoflavone). Calycosin, a core flavonoid component, has been shown to exert anti-PF effects via multiple targets. It has been demonstrated that the flavonoids present in the plant AM can alleviate alveolar inflammatory damage and reduce inflammatory cascade reactions. In addition, they have been shown to inhibit oxidative stress responses and protect AECs from oxidative damage. Furthermore, the flavonoids can inhibit the EMT process and block fibrosis-related signaling pathways such as TGF-*β*/Smad and PI3K/Akt/mammalian target of rapamycin (mTOR) (Ji et al., 2022, Liu et al., 2022).

### 3.2 *Astragalus* saponins

*Astragalus* saponins represent a key class of bioactive components in AM, with multiple derivatives isolated and identified—including astragaloside (AS) I–VIII, isoastragaloside, acetylastragaloside, and cycloastragenol. Among them, AS-IV is the key substance of this class and can intervene in the progression of PF through multiple targets. AS-IV exerts anti-PF effects by inhibiting inflammatory factor release and cell infiltration, reducing malondialdehyde levels via antioxidant activity, reversing EMT to decrease ECM deposition, and blocking the TGF-*β*/Smad pathway to suppress fibroblast-to-myofibroblast transformation (Jinjuvadia, Patel, Mamdani, & Liangpunsakul, 2013).

### 3.3 *Astragalus* polysaccharides (APS)

Polysaccharides are a class of naturally occurring, ubiquitously distributed macromolecules that have garnered considerable attention for their diverse biological and pharmacological activities. It has been demonstrated that APS, a constituent of the AM plant, possess a notable immunomodulatory effect. AM polysaccharides have been shown to regulate immune balance, promote macrophage polarization towards the M2 type, and block the NF-*κ*B signaling pathway, thereby inhibiting inflammatory storms. In addition, APS have been found to scavenge ROS and restore oxidative balance in the body. Furthermore, APS have been demonstrated to block the TGF-*β*/Smad signaling pathway, reducing ECM deposition and exerting antifibrotic effects (Jiang, Chen, Li, & Wang, 2023; Zhang, Xu, An, Sui, & Lin, 2020).

## 4 Mechanisms underlying anti-PF effects of bioactive constituents from AM

**Fig. 2:**
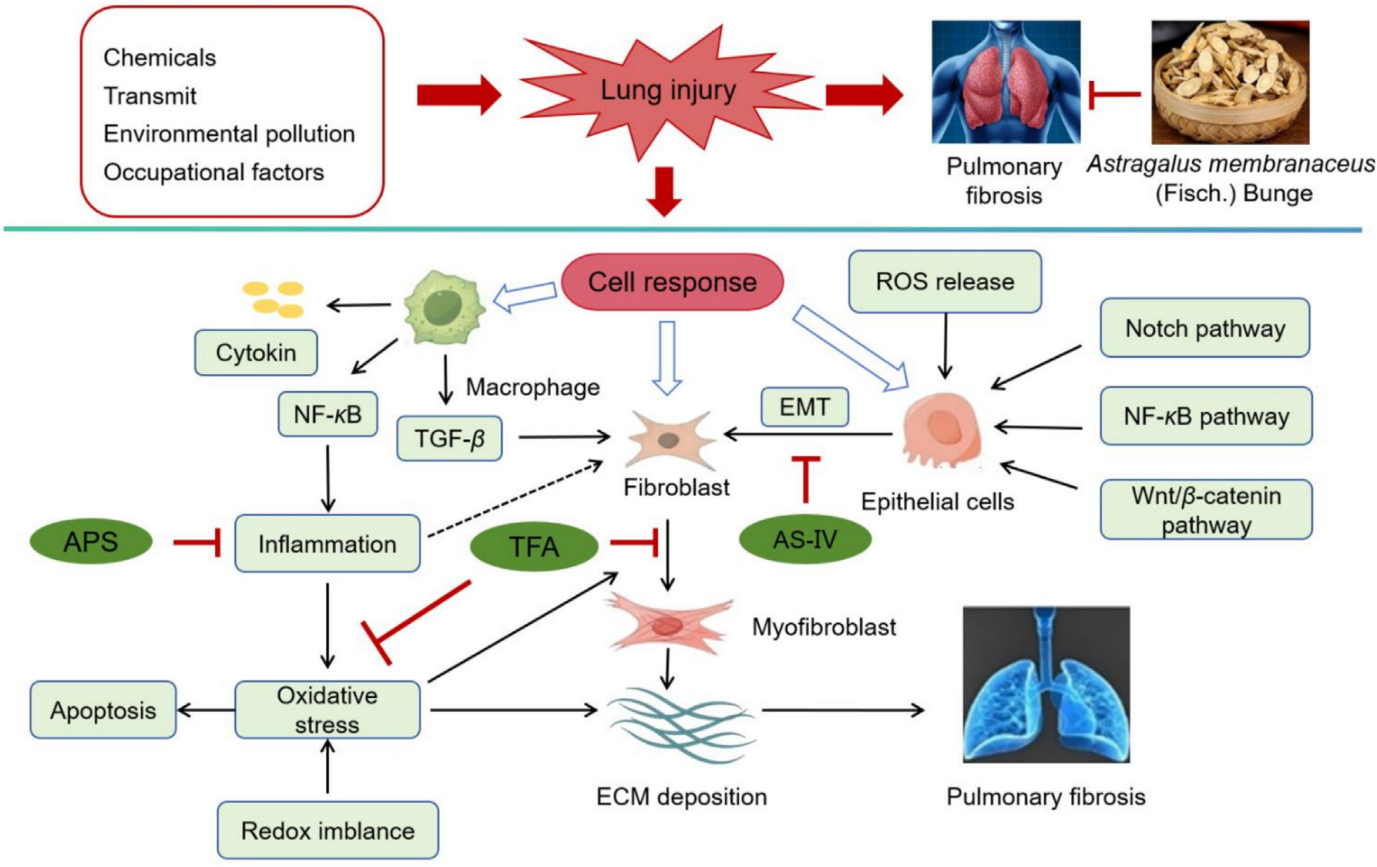
Mechanism of action of AM against PF.

EMT initiates the core pathological mechanism of PF, with inflammatory cascades acting as critical drivers and the accumulation of senescent fibroblasts playing critical roles in disease progression. This is accompanied by multidimensional pathological changes, including an imbalance in the lung-gut axis microbiota. The active components of AM work through a multi-component, multi-target, multi-pathway synergistic mode to inhibit EMT, clear senescent cells, alleviate inflammatory responses, block ferroptosis, and regulate the lung-gut axis. This multi-component, multi-target, and multi-pathway synergistic mode enables systematic intervention in the onset and progression of PF. As demonstrated in Fig. 2, the active components of AM, including astragalosides, flavonoids, and polysaccharides, exert anti-fibrotic effects in a synergistic manner by intervening in multiple key signaling pathways. The primary mechanisms encompass: Inhibition of the TGF-*β*/Smad and Wnt/*β*-catenin signaling pathways has been demonstrated to be efficacious in blocking EMT and the conversion of fibroblasts into myofibroblasts. Suppression of NF-*κ*B and NLRP3 inflammasomes has also been shown to reduce inflammatory responses, and promotion of the apoptosis of senescent fibroblasts has been shown to decrease ECM deposition. The mechanistic diagram under consideration integrates the multi-component, multi-target synergistic mode of AM, reflecting its systemic intervention advantages within the complex pathological network of PF (Zhu et al., 2022).

### 4.1 Suppression of EMT

In the context of PF, the inhibition of EMT has emerged as a pivotal mechanism in the research on AM, with robust support from both *in vitro* and *in vivo* studies. This finding encompasses a diverse array of active components, including AS-IV and total AM saponins. The precise regulation of EMT-related signaling pathways, the prevention of fibroblast activation and ECM deposition, and the slowing of disease progression are all effects of the drug.

Research findings indicate that the three primary active components of AM—Astragalus *flavonoids*, *Astragalus* saponins and APS*—are capable of exerti*ng a synergistic effect, thereby inhibiting EMT through the engagement of multiple targets and pathways. AS-IV, a pivotal regulator of intracellular signaling, reduces *α*-SMA protein expression in lung tissue by 41% compared to the model group via inhibiting the TGF-*β*1/Smad and PI3K/Akt/Forkhead box O3 (FOXO3a) pathways, reversing E-cadherin downregulation, directly blocking EMT, and ameliorating PF (Luo et al., 2025). Flavonoid compounds from AM, such as TFA, quercetin, and kaempferol, have been shown to regulate the TGF-*β*/Smad pathway or disrupt the follistatin-like 1 (FSTL1)-TGF-*β*1 positive feedback loop, significantly downregulating EMT-related transcription factors, such as Snail and Slug. This effect is further compounded by the presence of astragaloside compounds (Glancy, 2020, Lan et al., 2025).

**Fig. 3:**
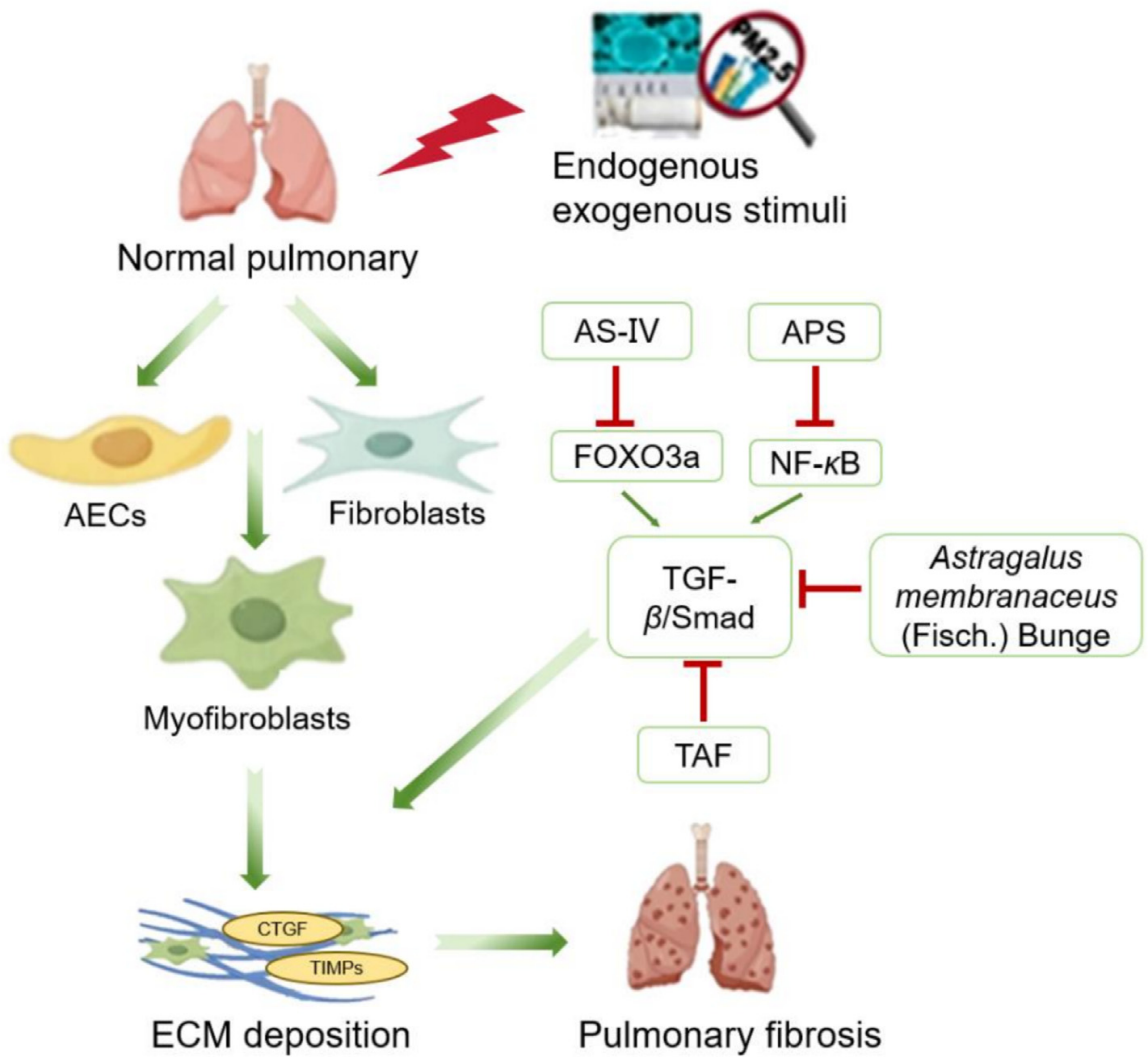
Main mechanism by which AM and its active ingredients regulate EMT. TIMPs: tissue inhibitors of metalloproteinases; CTGF: connective tissue growth factor.

Concurrently, APS reduces the expression of *α*-SMA and the secretion of collagen I by inhibiting the activation of the NF-*κ*B pathway, thereby alleviating the persistent inflammatory microenvironment that drives EMT (Wei, Qi, Liu, & Li, 2023). It is noteworthy that the combined application of AS-IV and quercetin provides direct evidence that this combination reduces inflammatory cell infiltration, enhances lung tissue structure, exerts a more pronounced therapeutic effect than either agent alone, and promotes autophagy to regulate key pathways, as demonstrated by a plethora of studies. (Luo et al., 2025). This effect is achieved by promoting autophagy, thereby regulating the key pathways involved in the disease pathology. This multi-level, complementary intervention—from signaling pathways to the disease microenvironment—forms the synergistic basis for AM to efficiently inhibit EMT. As shown in Fig. 3, harmful stimuli activate AECs and fibroblasts to differentiate into myofibroblasts, which could induce ECM deposition through EMT, ultimately leading to PF. AM and its bioactive components, including astragalosides, APS, and *Astragalus* flavonoids, exert anti-fibrotic effects by multi-targetedly inhibiting the TGF-*β*/Smad pathway, thereby blocking myofibroblast activation and ECM accumulation. Despite the robust body of evidence supporting this mechanism, there is currently a paucity of clinical evidence. EMT markers have only been detected in lung tissue samples from PF patients, and no interventional studies have confirmed that AM can directly inhibit the EMT process in humans. Consequently, it is impossible to establish a direct link from mechanistic research to clinical efficacy.

### 4.2 Regulating inflammatory cascade reactions and immune microenvironment

**Fig. 4:**
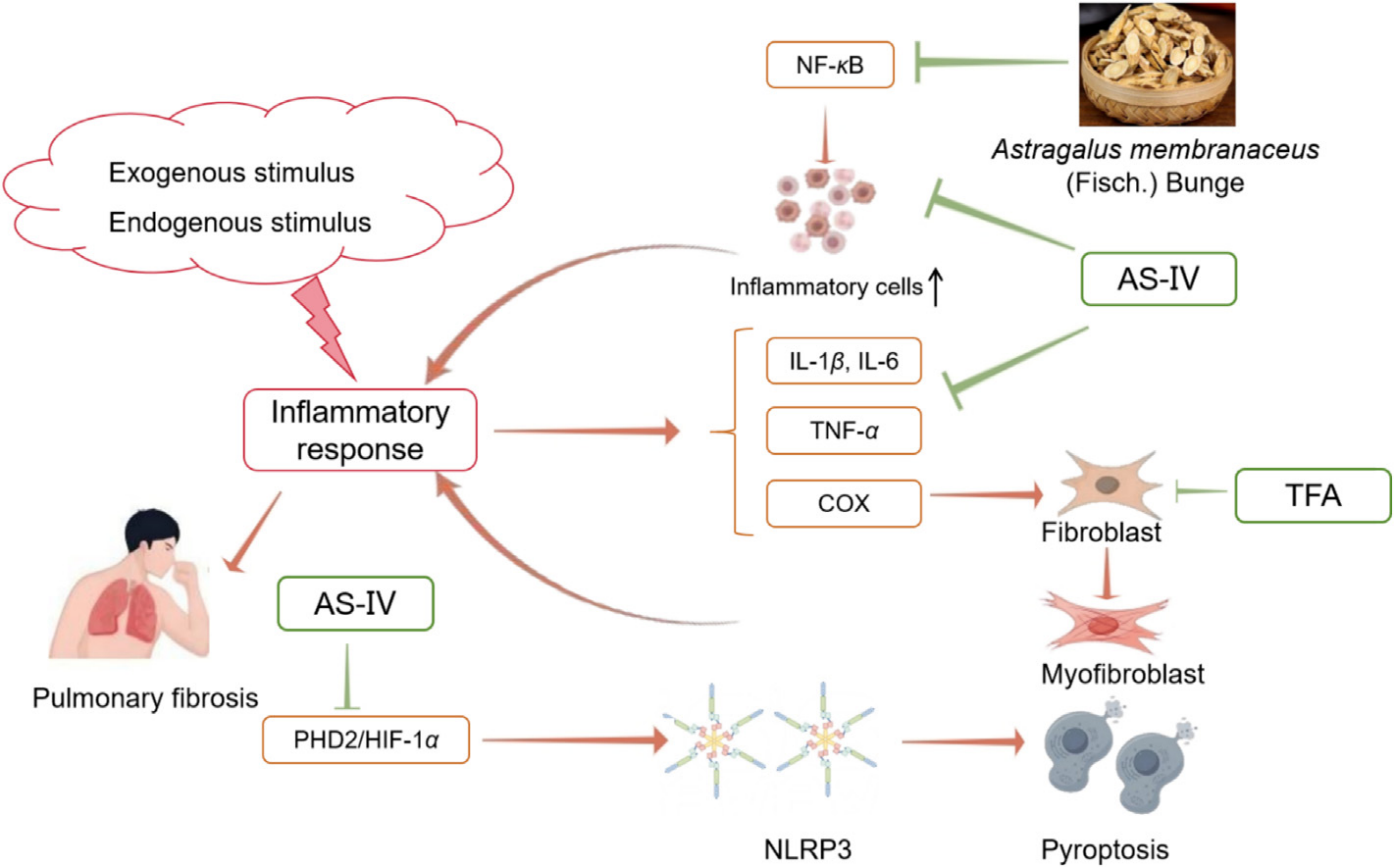
Main mechanism by which AM and its active components inhibit inflammatory response.

Persistent inflammation has been identified as a major trigger and driver of PF progression. The active components of AM work synergistically to achieve comprehensive coverage, from local inflammation suppression to systemic immune regulation. The evidence supporting this mechanism is relatively substantial. AS-IV has been demonstrated to inhibit the activation of the NLRP3 inflammasome through the prolyl hydroxylase domain protein 2 (PHD2)/HIF-1*α* pathway, thereby rapidly curbing the local inflammatory storm (Xi et al., 2023). It has been demonstrated that AS-IV and APS can effectively inhibit the Toll-like receptor 4/NF-*κ*B signaling pathway, reduce levels of inflammatory factors such as TNF-*α* and IL-6, and delay the progression of PF (Wei et al., 2023, Wu et al., 2021a). *In vitro* experiments have demonstrated that TFA have the capacity to alleviate immune dysregulation by regulating the infiltration of inflammatory cells and promoting macrophage polarization towards the M_2_ phenotype (Yang et al., 2023). As shown in Fig. 4, endogenous and exogenous stimuli trigger inflammatory responses by activating the NF-*κ*B pathway, which promotes inflammatory cell infiltration and the release of pro-inflammatory cytokines such as IL-1*β*, IL-6, and TNF-*α*. These factors drive fibroblast activation and PHD2/HIF-1*α*-NLRP3-mediated pyroptosis, thereby exacerbating PF. In AM, astragalosides inhibit NF-*κ*B activation and cytokine production while regulating pyroptotic signaling. *Astragalus* flavonoid derivatives (AFD) could block fibroblast activation, collectively exerting multi-target anti-fibrotic effects.

**Fig. 5:**
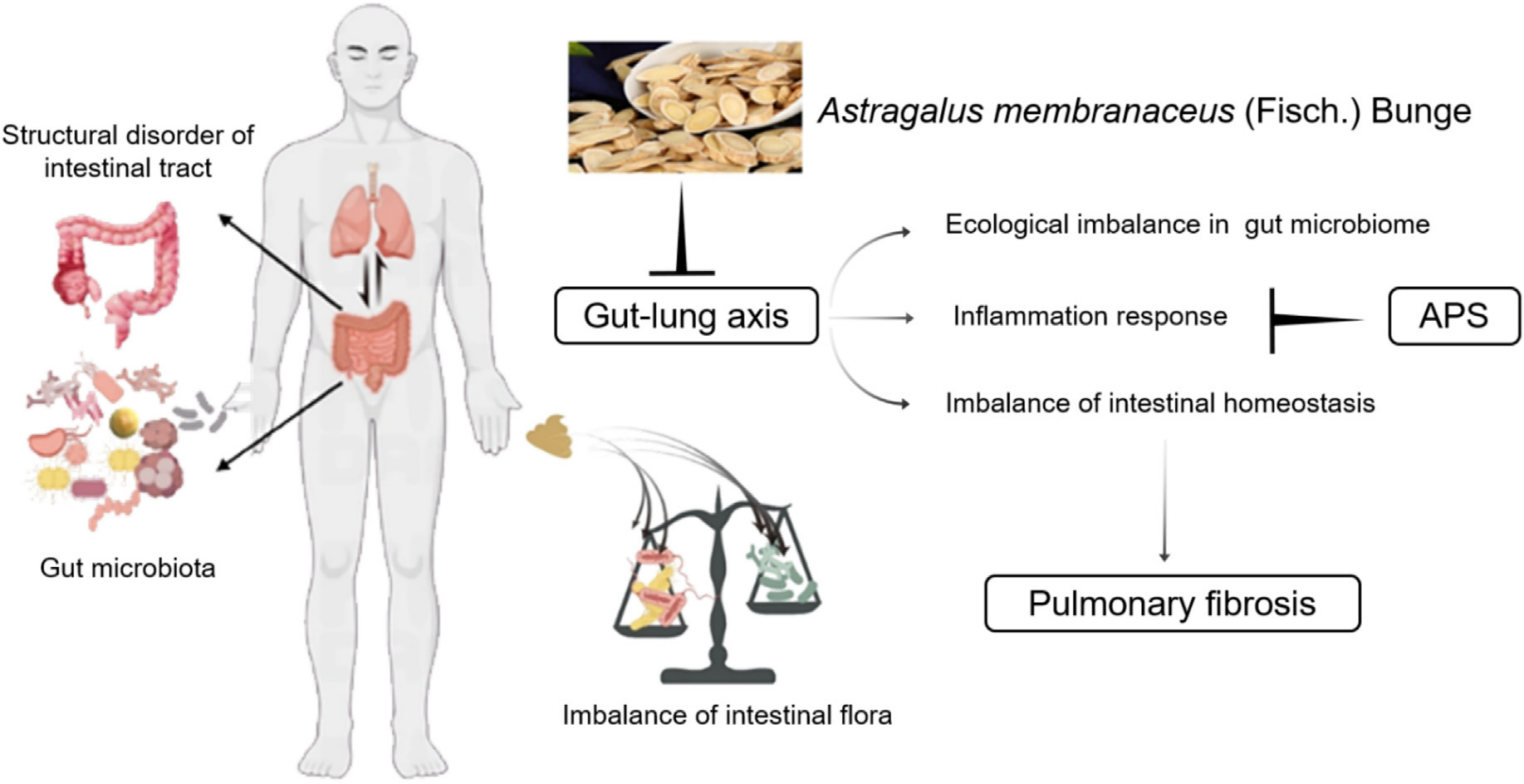
Main mechanism by which AM and its active ingredients regulate gut-lung axis.

Moreover, *in vivo* studies have indicated that APS can modulate the lung-gut axis, increase the relative abundance of beneficial bacteria such as *Lactobacillus* and *Akkermansia*, enhance immunity and the balance of the gut microbiota, and suppress systemic inflammatory responses, thereby engendering a favourable systemic immune environment for local lung repair (Wei, Qi, Liu, & Li, 2023). The synergistic effect of rapid local anti-inflammation and systemic immune homeostasis regulation effectively disrupts the vicious cycle of inflammation-fibrosis. As shown in Fig. 5, gut microbiota dysbiosis triggers intestinal microecological imbalance, inflammatory responses, and disruption of intestinal homeostasis via the gut-lung axis, thereby driving the progression of PF. APS can inhibit gut-lung axis-mediated inflammation, ameliorate gut microbiota dysbiosis and homeostatic disturbance, block pathological signal transmission from the gut to the lungs, and exert anti-fibrotic effects.

### 4.3 Regulation of ferroptosis and senescent cell death

Ferroptosis and the accumulation of senescent cells associated with oxidative stress are important features of PF (Almalki & Almujri, 2024). The active components of AM have been shown to exhibit significant pathway cross-talk and synergistic effects in combating these pathological processes. Recent studies have confirmed the ability of quercetin to activate Sirtuin 1 (Sirt1)/nuclear factor erythroid 2-related factor 2 (Nrf2)/glutathione peroxidase 4 (GPX4), thereby inhibiting ferroptosis. Similarly, AS-IV has been shown to inhibit ferroptosis through the Nrf2/solute carrier family 7 member 11 (SLC7A11)/GPX4 axis, with the additional benefits of restoring intracellular iron transport and storage, enhancing cellular antioxidant capacity, reversing tissue damage caused by ferroptosis, and improving PF. The augmentation of GPX4 activity in lung tissue by up to 50% has been demonstrated to enhance antioxidant defense and synergistically inhibit ferroptosis (Wang et al., 2022, Wen et al., 2024).

The abnormal accumulation of senescent fibroblasts is a key driving force in the development of PF. The senescence-associated secretory phenotype (SASP) they secrete has been shown to create a persistent pro-fibrotic and pro-inflammatory microenvironment through a paracrine mechanism, thereby driving the activation and proliferation of normal fibroblasts. Furthermore, senescent cells themselves possess anti-apoptotic properties, allowing them to survive long-term in lung tissue and become a major source of continuous ECM production, ultimately exacerbating the fibrosis process. Consequently, therapeutic interventions that reduce the senescent cell burden can retard the progression of PF and other age-related diseases. Flavonoid components from AM, such as quercetin, have been shown to promote the apoptosis of senescent fibroblasts and delay the progression of PF by upregulating the expression of caveolin-1 and Fas proteins (Hohmann, Habiel, Coelho, Verri, & Hogaboam, 2019). Despite the absence of direct evidence for the presence of saponins and polysaccharides in this process, it is hypothesized that these substances may indirectly facilitate the elimination of senescent cells by improving the microenvironment through anti-inflammatory and antioxidant effects.

## 5 Mechanism of action of multiple active components of AM in anti-PF through multi-targets and multi-pathways

The primary benefit of AM in the treatment of PF is attributable to the ‘complementary functions, overlapping targets, and synergistic pathways’ network of its three primary active components: saponins, flavonoids, and polysaccharides. Through the precise targeting of their respective core targets and the interweaving of their action pathways, it has been demonstrated to effectively improve PF. The molecular mechanisms of active components from AM against PF are shown in Table 1.

**Table 1:** Molecular mechanisms of active components from AM against PF.

Mechanisms	Models	Effects and mechanisms	Components	References
Suppression of EMT	Male C57BL/6 mice	Inhibition of TGF-*β*/Smad signaling pathway transduction	AS-IV; *Astragalus* flavonoids; Chalcone	Zhang et al., 2020, Xiao et al., 2023b
	Male C57BL/6 mice	Inhibition of NF-*κ*B signaling pathway transduction	APS	Zhang, Xu, An, Sui, & Lin, 2020
	BEAS-2B human bronchial epithelial cells (BEAS-2B); normal human lung fibroblasts; male C57BL/6J mice	Inhibition of fibroblast-to-myofibroblast conversion	AS-IV	Zhang et al., 2023
		Reducing ECM deposition	Quercetin; kaempferol	Zhang et al., 2024
Promotion of senescent fibroblast death	Human lung adenocarcinoma epithelial cell line A549 (A549)	Modulating caveolin-1 and Fas expression, promoting Akt activation, increasing sensitivity of senescent fibroblasts to apoptosis	Quercetin	Hohmann et al., 2019
Reducing the inflammatory cascade	Male Sprague-Dawley rats	Reducing expression levels of inflammatory factors	AS-IV; TFA; APS	Wu et al., 2021b, Yang et al., 2023, Ming et al., 2022
Inhibition of oxidative stress	Nrf2-deficient (Nrf2^−/−^) mice	Reducing ROS expression levels and inhibiting oxidative stress	Quercetin	Boots et al., 2020
Regulation of autophagy	C57BL/6N male mice	Inhibiting mTOR-mediated autophagy	AS-IV	Li et al., 2025
Inhibition of ferroptosis	C57BL/6J male mice	Inhibiting iron death by activating HIF-1*α*-EGFR signaling pathway, reducing collagen deposition	AS-IV; APS	Wang et al., 2022, Lee et al., 2024
Regulating gut-lung axis and increasing beneficial intestinal bacteria	C57BL6 male mice	Increasing abundance of beneficial intestinal flora and restoring intestinal homeostasis	APS; quercetin	Wei, Qi, Liu, & Li, 2023

### 5.1 NF-κB pathway

It has been demonstrated that polysaccharides have the capacity to inhibit the activation of the NF-*κ*B pathway and reduce the release of inflammatory factors such as TNF-*α* and IL-6. Flavonoids have been demonstrated to regulate NF-*κ*B-mediated macrophage polarization, while saponins have been shown to inhibit NLRP3 inflammasome activation through PHD2/HIF-1*α*-NF-*κ*B cross-regulation (Wei et al., 2023, Xi et al., 2023, Yang et al., 2025), The three work in synergy to reduce the inflammatory cascade response and block the initiation of the inflammation-fibrosis process.

### 5.2 Nrf2 antioxidant pathway

Quercetin activates Nrf2 through Sirt1, while AS-IV directly upregulates Nrf2 nuclear translocation. The two elements work in a synergistic manner to enhance the activity of antioxidant enzymes, such as GPX4 and superoxide dismutase (SOD). Polysaccharides have been shown to reduce the inhibitory effect of oxidative stress on the Nrf2 pathway by scavenging ROS (Jiang, Chen, & Wang, 2023), thereby forming a synergistic antioxidant network of activation-enhancement-protection. In summary, astragaloside saponins, flavonoids, and polysaccharides have been shown to exert anti-PF effects by blocking the core pathological pathways of PF, thereby establishing a multi-level and networked therapeutic system.

This synergistic action overcomes the limitations of single chemical drugs, effectively intervening in the vicious cycle of ‘injury-inflammation-fibrosis’ by mimicking the body’s complex repair regulatory networks. Studies have shown that AM injection can downregulate pulmonary Jagged1/Notch1 and attenuate BLM-induced PF. Notably, other studies found that the combined application of astragalosides and flavonoids did not exhibit synergistic effects, indicating that the synergistic activities of different component combinations are specific; thus, further screening for optimal compatibility ratios is required (Zhou, Liao, Zhang, Wang, & Wan, 2016). This study provides direct pharmacological evidence for the synergistic anti-fibrotic effects of multiple components in AM. Regarding the synergistic interaction between astragalosides and flavonoids, *in vitro* and *in vivo* experiments confirmed that the combined use of astragalosides and quercetin synergistically enhances autophagic activity, manifested as upregulated expression of microtubule-associated protein 1A/1B-light chain 3 (LC3) and Beclin-1 and downregulated p62. Meanwhile, this combination synergistically inhibits the expression of pyroptosis-related proteins, including NLRP3, Caspase-1, IL-1*β*, and IL-18, thereby cooperatively attenuating the EMT process in AECs (Luo et al., 2025). This study reveals the molecular mechanism underlying the synergistic anti–fibrotic effects of different bioactive components in AM from the perspective of crosstalk regulation between autophagy and pyroptosis.

Network pharmacology analyses indicate that multiple active constituents of AM, including quercetin, kaempferol, daidzein, and canavanine, jointly target 97 PF-associated genes such as *PTGS2*, *MAPK1*, *MAPK14*, and *TGF-β*, and synergistically modulate multiple signaling pathways including advanced glycation end products-receptor for advanced glycation end products (AGE-RAGE) and IL-17 (Li, He, Zhou, & Deng, 2022). Collectively, these findings demonstrate that distinct bioactive components of AM exert network-based regulation by acting on multiple targets and pathways, warranting further mechanistic investigation into their synergistic potentiation (Li, He, Zhou, & Deng, 2022).

This finding provides substantial evidence for the ‘multiple components-multiple targets-multiple pathways’ hypothesis, and offers significant mechanistic support for the clinical application of AM, as well as for the development of innovative compound formulations.

## 6 Limitations of current study

Despite the wealth of evidence from preceding studies on the effects of AM on PF, the current body of evidence has significant limitations, which seriously hinders its clinical translation. The field of translational medicine aims to translate scientific discoveries into practical therapeutic strategies, yet this bench-to-bedside transition is often fraught with substantial challenges. Current evidence is predominantly derived from *in vitro* cellular assays and *in vivo* animal studies, with a severe paucity of high-quality clinical trials. While cellular assays primarily predict potential targets and mechanisms, the BLM-induced PF mouse model—the most widely used preclinical evaluation system—exhibits notable limitations (Schönke & Rensen, 2024; Zhang et al., 2025a). Firstly, this model fails to fully recapitulate the chronic progressive nature of human PF. Fibrosis typically undergoes spontaneous resolution within weeks post-injury, significantly differing from the persistent progression observed in human pathology (Moeller, Ask, Warburton, Gauldie, & Kolb, 2008). Secondly, while conventional biochemical quantification of total hydroxyproline content serves as the “gold standard” for assessing fibrosis severity, residual collagen degradation fragments that are not fully cleared during the spontaneous resolution phase may lead to an “intracellular hydroxyproline footprint”, thereby causing misinterpretation of the true fibrotic status (Song et al., 2022). Furthermore, interspecies differences compromise the reliability of predictions regarding drug efficacy and toxicity. In recent years, the establishment of humanized mouse models and tree shrew models has provided novel strategies to overcome interspecies differences. Tree shrews exhibit closer genetic, anatomical, and metabolic similarities to humans, making them promising ideal animal models for investigating the pathogenesis of PF (Larson-Casey et al., 2020).

To date, no well-designed randomized controlled trials (RCTs) have validated AM’s efficacy in PF, its proposed therapeutic potential relies primarily on preclinical data and TCM clinical experience, necessitating rigorous clinical validation. Consequently, the design of clear and reliable clinical trials constitutes a complex task. Furthermore, despite the well-documented preclinical anti-PF activity of AM and its active components, their unfavorable pharmacokinetic profiles (e.g., poor oral bioavailability) severely hinder clinical translation. Research has demonstrated that the primary active constituent of AM, astragaloside, exhibits remarkably low oral bioavailability, with an absolute bioavailability of a mere 2.2% in rats. This phenomenon can be attributed to various factors, including poor intestinal permeability, high molecular weight, low lipophilicity, and significant hepatic first-pass effect (Gu et al., 2004). This results in inadequate systemic exposure in the body, which hinders the achievement of effective therapeutic concentrations in the target organs.

To address the low bioavailability of AM’s bioactive components, research has shifted toward formulation technology innovations to overcome these limitations, including poor solubility, low permeability, and pronounced first-pass metabolism. For instance, encapsulating APS in liver- and intestine-targeted nanoparticles has been shown to facilitate active delivery to the lungs, thereby increasing the accumulation of key active substances in this region (Gu et al., 2004, Liu et al., 2025). The design of prodrugs or phospholipid complexes has been demonstrated to enhance intestinal absorption (Yu et al., 2024). The advancement of AM formulations from traditional bulk materials to innovative formulations based on clearly defined active components and optimised pharmacokinetics for precise delivery is a key approach to ensuring controllable efficacy and reliable safety. These strategies provide feasible technical pathways for the clinical translation of astragalosides. However, systematic pharmacokinetic-pharmacodynamic (PK-PD) correlation studies are still required to define the optimal delivery strategies and clarify their advantages in the treatment of PF.

The accelerated growth of the TCM industry has concomitantly given rise to an increase in the number of low-quality and counterfeit herbal products (Zhu, Liu, Qiu, Dai, & Gao, 2022). It is evident that inconsistencies in the quality of AM are attributable to variations in terrain, climate, and other ecological factors (Dong et al., 2024). Furthermore, the processing methods employed have been shown to exacerbate the fluctuations in the content of active ingredients, thereby compromising the reproducibility of research results and the reliability of clinical applications.

The therapeutic effects of anti-PF drugs pirfenidone and nintedanib are limited and vary among individuals (Provenzani et al., 2025). It has been demonstrated that AM and its active components possess a multi-pathway anti-inflammatory, anti-fibrotic, and immunomodulatory effect (Pan et al., 2023, Tu et al., 2024). These effects may complement or synergies with existing drugs through pathways such as PI3K/Akt and TGF-*β*/Smad. Furthermore, AM has demonstrated hepatoprotective, gastrointestinal regulatory, and immunomodulatory properties in traditional applications (Wang et al., 2024, Zheng et al., 2024). The combination of these agents may enhance the anti-PF effects via multi-target regulation, reduce the adverse effects of targeted drugs, and improve patient adherence and quality of life. However, AS-IV has a protein binding rate of approximately 90%, meaning it extensively binds to plasma proteins (Zhang, Zhu, Chen, & Du, 2007). Combined use of these medications may result in alterations to the plasma concentrations of nintedanib and pirfenidone, which could potentially lead to fluctuations in efficacy or an increased risk of toxicity. Systematic studies on pharmacokinetic and pharmacodynamic interactions are still lacking. In order to verify the feasibility of such interactions, rigorous preclinical research and well-designed clinical trials are required.

## 7 Future prospects

A substantial body of research has been conducted on the active components of AM, and new therapeutic targets for PF have been identified. These developments offer the potential for a significant improvement in the treatment of patients with PF, and in their quality of life. Nevertheless, the pathway to achieving these advancements is not without challenges. In order to transform the anti-PF therapeutic potential of TCM AM into viable clinical treatment, in-depth research will be required in the future.

To promote the transformation of AM and its active components from empirical use to a data-driven intelligent TCM model, future research can leverage machine learning algorithms and omics data to systematically analyze the synergistic interaction network of AM’s multi-components, multi-targets, and multi-pathways, and predict the action patterns of its core targets associated with PF. Deep learning models can simulate the dynamic interactions between AM’s active components and the lung tissue microenvironment, assisting in the screening of optimal component combinations and dosage ratios. In addition, AI-driven drug design can optimize the structures of AM components with low bioavailability and accelerate the development of targeted delivery systems (Guo et al., 2024).

It is imperative that clinical translation adheres to internationally standardized drug development pathways. In the future, efforts should be made to promote clinical trials of standardized AM and its active component preparations, investigate appropriate dosages and safety, conduct randomized controlled trials, and explore biomarkers that reflect multi-pathway regulatory effects, providing an objective basis for efficacy evaluation.

The quality of pharmaceuticals is pivotal in ensuring the reproducibility of their efficacy. The establishment of a dual-indicator quality control standard for AM medicinal materials based on ‘chemical fingerprinting-bioactivity evaluation’ is a primary task. On the one hand, techniques such as high-performance liquid chromatography (HPLC) and ultra-performance liquid chromatography-tandem mass spectrometry (UPLC-MS/MS) are utilised to ascertain the content thresholds of polysaccharides, saponins, and flavonoid active components in AM from divergent origins, thereby constructing characteristic chemical fingerprints (Champati et al., 2024). On the other hand, in conjunction with lung fibrosis cell models, a bioactivity evaluation system has been instituted to ensure that the medicinal material not only meets component standards but also possesses clear anti-fibrotic activity (Li et al., 2023). Concurrently, standardised processing techniques and extract production procedures are formulated to provide a foundation for commercial production.

Translating the anti-PF potential of AM into clinical reality is a complex system project that requires deep interdisciplinary integration. It is imperative for future researchers to explore the molecular mechanisms in depth and combine studies of pharmacokinetics, pharmaceutics, clinical research design, and quality standards. By following comprehensive and rigorous modern scientific research strategies, the scientific value of this traditional Chinese medicinal treasure can be unlocked, thereby providing PF patients with a new therapeutic option that embodies Chinese wisdom, proven efficacy, and controllable quality.

## 8 Conclusion

PF is distinguished by its insidious onset and intricate etiology. At present, the majority of prevalent clinical treatments (e.g. pirfenidone and nintedanib) are classified as single-target drugs, which characteristically exhibit limited efficacy, discernible side effects, and considerable treatment costs. TCM has demonstrated considerable efficacy in the treatment of PF, with its primary advantage lying in the ‘multi-component, multi-target, multi-pathway’ synergistic intervention mechanism. In the theoretical framework of TCM, PF is closely associated with conditions such as ‘*fei bi*’, ‘*fei wei*’, and ‘*fei yong*’ (lung abscess). The primary approach to clinical syndrome differentiation and treatment involves the notification of the lungs and kidneys, the strengthening of the spleen, and the promotion of blood circulation and the resolution of stasis (Fabre and Nicholson, 2020, Hosseini et al., 2018, Martusewicz-Boros and Górska, 2020).

AM, a TCM herb known for tonifying *qi* and nourishing blood, has been shown to have remarkable efficacy in improving PF. Saponins, flavonoids and polysaccharides have been shown to interact synergistically through a multi-component, multi-target, multi-pathway mechanism to combat PF. This intervention model overcomes the limitations of single-target drugs that attack only one point, and it also achieves multi-step synchronous regulation of the ‘injury-inflammation-fibrosis’ vicious cycle. In addition, it restores body homeostasis through complementary targets, pathway crosstalk, and local-systemic synergy. This reflects the modern scientific connotation of the TCM concepts of ‘holistic view’ and ‘principle of harmonization’. Among these, polysaccharides, flavonoids, and saponins have been shown to inhibit the nuclear NF-*κ*B pathway, thereby reducing inflammatory responses. In addition, these compounds have been observed to scavenge ROS and restore redox balance. Furthermore, they have been demonstrated to block the TGF-*β*/Smad signaling pathway, thus decreasing ECM deposition and exerting anti-fibrotic effects. This multi-pathway intervention strategy, targeting the core pathological processes of PF (inflammatory response, oxidative stress, and tissue remodeling), overcomes the limitations of single-target drugs and helps restore lung tissue homeostasis, providing a more fundamental and sustainable approach for clinical treatment. Given the complex pathogenesis of PF, the scarcity of effective therapeutic drugs, and the significant socioeconomic burden brought about by the continuous rise in PF incidence after the COVID-19 pandemic, the development of highly efficient, low-toxicity, and economical PF treatment drugs is of urgent and significant socioeconomic importance. The therapeutic effects of AM on PF have been partially studied, and it is rich in a variety of active components with anti-inflammatory, antioxidant, anti-aging, and other pharmacological effects, laying an important foundation for the development of innovative drugs based on its unique synergistic mechanism.

To further advance the clinical translation and mechanistic research of AM in PF treatment, several strategic recommendations are proposed. In terms of clinical trial design, multicenter, large-sample, long-term follow-up randomized controlled trials (RCTs) are warranted to define the optimal dosage, treatment course, and applicable populations for individual AM components. Concurrently, objective evaluation criteria, including pulmonary function parameters and imaging characteristics, should be integrated to rigorously validate clinical efficacy and safety. Regarding biomarker development, specific biomarkers closely associated with the anti-fibrotic effects of AM (e.g., inflammatory cytokines and fibrosis-related proteins) need to be screened and validated, providing precise references for therapeutic evaluation, disease monitoring, and personalized therapy. For quality control standards, accurate quantitative assays for key bioactive components (astragalosides, flavonoids, and polysaccharides) should be established. Furthermore, the whole-process quality control covering cultivation, extraction, purification, and pharmaceutical manufacturing must be standardized to ensure efficacy stability and batch consistency. Mechanistically, integrated omics technologies (genomics, transcriptomics, metabolomics) combined with artificial intelligence (AI) algorithms can be utilized to systematically uncover potential targets and interactive network pathways. This approach will further elucidate the synergistic ‘multi-component, multi-target, multi-pathway’ anti-fibrotic molecular mechanism, thereby laying a solid scientific foundation for innovative drug development derived from AM.

Integrating AM’s TCM theoretical basis, its active components’ pharmacological effects, and anti-PF scientific evidence to develop highly effective, low-toxic, and cost-efficient innovative drugs is expected to mitigate or even reverse the high post-pandemic PF incidence, generating substantial social and economic benefits.

## CRediT authorship contribution statement

**Xiujuan Sun:** Writing – original draft. **Guangyue Su:** Writing – review & editing. **Jingwen Zhao:** Supervision. **Yiteng Ma:** Validation. **Zhihao Kong:** Validation.

## Declaration of competing interest

The authors declare that they have no known competing financial interests or personal relationships that could have appeared to influence the work reported in this paper.

## Appendix A Supplementary data

Supplementary Data 1 This supplementary file provides a basic dataset of Astragalus membranaceus chemical constituents, including the types, structures, and identification evidence of its flavonoids, saponins, and polysaccharides, as core supporting data for its pharmacological studies, quality control, and resource development.
