# Supplementary material for: Combatting pulmonary fibrosis with Astragalus membranaceus: A review of active components and multifaceted mechanisms
Source: Chin Herb Med. 2026 May 1;18(3):541–54. doi: 10.1016/j.chmed.2026.04.007 (PMC13389998; doi:10.1016/j.chmed.2026.04.007)
Supplement: Supplementary Data 1 — This supplementary file provides a basic dataset of Astragalus membranaceus chemical constituents, including the types, structures, and identification evidence of its flavonoids, saponins, and polysaccharides, as core supporting data for its pharmacological studies, quality control, and resource development. [file mmc1.docx]

**Supplementary materials**

**Table S1** Chemical constituents in *Astragalus* flavonoid compounds.

| No. | Names | Molecular formula | Species | Parts | Appraisal | References |
| --- | --- | --- | --- | --- | --- | --- |
| F1 | Quercetin | C_15_H_10_O_7_ | AM | Leaves | Spectral analysis | Wang et al., 2017 |
| F2 | Quercetin 3‐*O*‐*β*‐*D*‐glucopyranoside | C_21_H_20_O_12_ | AM | Aerial parts, leaves | Spectral analysis | Wang, Zhu, Chen, Yu, & Ma, 2017 |
| F3 | Astraflavonoid A | C_36_H_36_O_18_ | AM | Stems | Chemical and spectroscopic analysis | Hao et al., 2016 |
| F4 | Kaempferol | C_15_H_10_O_6_ | AM | Roots | Chemical and spectroscopic analysis | Bian & Li, 2008 |
| F5 | Kaempferol 3‐*O*‐*β*‐*D*‐glucoside | C_21_H_20_O_11_ | AM | Stems | Chemical and spectroscopic analysis | Hao, Li, Li, Liu, & Ruan, 2016 |
| F6 | Kaempferol 3‐*O*‐(2‐*O*‐*α*‐*L*‐rhamnopyranosyl)‐*β*‐*D*‐glucopyranoside | C_27_H_30_O_16_ | AM | Stems | Chemical and spectroscopic analysis | Hao, Li, Li, Liu, & Ruan, 2016 |
| F7 | Rhamnocitrin 3‐*O*‐neohesperoside | C_28_H_32_O_15_ | AM | Stems | Chemical and spectroscopic analysis | Hao, Li, Li, Liu, & Ruan, 2016 |
| F8 | Kaempferol 3,7‐di‐*O*‐*β*‐*D*‐glucopyranoside | C_27_H_30_O_16_ | AM | Stems | Chemical and spectroscopic analysis | Hao, Li, Li, Liu, & Ruan, 2016 |
| F9 | Astraflavonoid B | C_43_H_48_O_23_ | AM | Stems | Chemical and spectroscopic analysis | Hao, Li, Li, Liu, & Ruan, 2016 |
| F10 | Isorhamnetin | C_16_H_12_O_7_ | AM | Roots | Spectroscopy | Bian & Li, 2008 |
| F11 | Quercetin3‐*O*‐*β*‐*D*‐neospheroside (Rutin) | C_27_H_30_O_16_ | AM | Stems | Spectroscopy | Bian & Li, 2008; Wen, Cheng, Zheng, Huang, & Han, 2010 |
| F12 | Isorhamnetin3‐*O*‐*β*‐*D*‐glucoside | C_22_H_22_O_12_ | AM | Aerial part | Spectral analysis | Bi et al., 2007 |
| F13 | Kaempferol 4'‐methoxyl‐3‐*O*‐*β*‐*D*‐glucoside | C_22_H_22_O_11_ | AM | Aerial part | Spectral analysis | Bi, Yu, Li, Lin, & Gao, 2007 |
| F14 | Oroxylin-A | C_16_H_12_O_5_ | AM | Roots | Chemical and spectroscopic analysis | Tian et al., 2016 |
| F15 | Wogonin | C_16_H_12_O_5_ | AM | Roots | Chemical and spectroscopic analysis | Tian, Deng, Zhou, & Cong, 2016 |
| F16 | 4',7-Dihydroxyflavone | C_15_H_10_O_4_ | AM | Roots | NMRS | Li et al., 2017 |
| F17 | 3',4',7‐Trihydroxy flavone | C_15_H_10_O_5_ | AM | Roots | NMRS | Li, Li, & Yan, 2017 |
| F18 | Rhamnocitrin 3‐*O*‐*β*‐*D*‐glucopyranoside | C_28_H_32_O_16_ | AM | Leaves; stems | Chemical and spectroscopic analysis | Hao, Li, Li, Liu, & Ruan, 2016; Wang, Zhu, Chen, Yu, & Ma, 2017 |
| F19 | Complanatuside | C_28_H_32_O_16_ | AM | Leaves; aerials parts, stems | Chemical and spectroscopic analysis | Bi et al., 2007; Bian & Li, 2008; Hao, Li, Li, Liu, & Ruan, 2016 |
| F20 | Rhamnocitrin 3‐*O*‐*β*‐*D*‐glucopyranoside (1 → 2) ‐*β*‐*D*‐apiofuranosyl | C_27_H_30_O_15_ | AM | Leaves | Spectroscopy | Wang et al., 2017 |
| F21 | Rhamnocitrin 3‐*O*‐*β*‐neohesperidoside | C_28_H_32_O_16_ | AM | Stems | Spectroscopy | Hao, Li, Li, Liu, & Ruan, 2016; Wang, Zhu, Chen, Yu, & Ma, 2017 |
| F22 | Tiliroside | C_30_H_26_O_13_ | AM | Leaves | Spectroscopy | Wang, Zhu, Chen, Yu, & Ma, 2017 |
| F23 | IsorhamnEchinatinetin 3‐*O*‐(6‐*O*‐*α*‐*L*‐fucopyranosyl)‐*β*‐*D*‐galactopyranoside | C_28_H_32_O_16_ | AM | Leaves | UHPLC-MS/MS | Du et al., 2024 |
| F24 | 6''‐Acetyl‐ononin | C_24_H_24_O_10_ | AM | Roots | HPLC-ELSD | Zhang et al., 2011 |
| F25 | Formononetin | C_16_H_12_O_4_ | AM | Roots | Spectroscopy | Bian et al., 2006; Wang, Zhu, Chen, Yu, & Ma, 2017 |
| F26 | Formononetin 7‐*O*‐*β*‐*D*‐glucoside (Ononin) | C_22_H_22_O_9_ | AM | Roots | Spectroscopy | Bian et al., 2006 |
| F27 | Calycosin | C_16_H_12_O_5_ | AM | Roots | Spectroscopy | Bian, Guan, Bi, Song, & Li, 2006; Bian & Li, 2008; Lee et al., 2023; Wang, He, & Luo, 1989 |
| F28 | Calycosin 7‐*O*‐*β*‐*D*‐(6‐*O*‐acetyl)‐glucopyranoside | C_24_H_24_O_11_ | AM | Roots | HPLC-ELSD | Xiao et al., 2009; Zhang, Liu, Hsiao, Kuo, & Lee, 2011 |
| F29 | Genistein | C_15_H_10_O_5_ | AM | Leaves; roots, stems | Chemical and spectroscopic analysis | Hao, Li, Li, Liu, & Ruan, 2016; Li et al., 2007; Wang, Zhu, Chen, Yu, & Ma, 2017 |
| F30 | Genistin | C_21_H_20_O_10_ | AM | Leaves; roots | Spectroscopy | Wang et al., 2017 |
| F31 | Glycitein | C_16_H_12_O_5_ | AM | Leaves | Spectroscopy | Wang, Zhu, Chen, Yu, & Ma, 2017 |
| F32 | Glycitin | C_22_H_22_O_10_ | AM | Leaves | Spectroscopy | Wang, Zhu, Chen, Yu, & Ma, 2017 |
| F33 | 3',7-Dihydroxy-5'-methoxyisoflavone | C_16_H_12_O_5_ | AM | Roots | Spectroscopy | Wen, Cheng, Zheng, Huang, & Han, 2010 |
| F34 | 4',5,7-Trihydroxy-3'- methoxyisoflavone | C_16_H_12_O_6_ | AM | Roots | Spectroscopy | Li, Zhou, Qiao, Fu, & Pei, 2007 |
| F35 | Pratensein 7-*O*-*β*-*D*-glucopyranoside | C_22_H_22_O_11_ | AM | Roots | − | Luo et al., 2012; Ma et al., 2005 |
| F36 | Sissotrin | C_22_H_22_O_10_ | AM | Roots | Spectroscopy | Zhang et al., 2012 |
| F37 | Sophorabioside | C_27_H_30_O_14_ | AM | Stems | HPLC | Hao, Li, Li, Liu, & Ruan, 2016 |
| F38 | Pendulone | C_17_H_16_O_6_ | AM | Roots | NMRS | Li, Li, & Yan, 2017 |
| F39 | (3*R*)-8,2'-Dihydroxy-7,4'-dimethoxyisoflavan | C_17_H_18_O_5_ | AM | Roots | HPLC-ELSD | Bian, Guan, Bi, Song, & Li, 2006; Li, Zhou, Qiao, Fu, & Pei, 2007; Song et al., 1997 |
| F40 | Isomucronulatol | C_17_H_18_O_5_ | AM | Roots | HPLC-ELSD | Zhang, Liu, Hsiao, Kuo, & Lee, 2011 |
| F41 | Isomucronulatol-7-*β*-*O*-glucoside | C_23_H_28_O_10_ | AM | Roots | HPLC-ELSD | Zhang, Liu, Hsiao, Kuo, & Lee, 2011 |
| F42 | 4,4',6'-Trihydroxychalcone | C_15_H_12_O_4_ | AM | Roots | Spectroscopy | Wang et al., 2014 |
| F43 | 4-Methoxy-4',6'-Dihydroxychalcone | C_16_H_14_O_4_ | AM | Roots | Spectroscopy | Wang, Wang, Ao, Dai, & Na, 2014 |
| F44 | 4,4'-Dimethyl-6'-hydroxy chalcone | C_17_H_16_O_2_ | AM | Roots | Spectroscopy | Wang, Wang, Ao, Dai, & Na, 2014 |
| F45 | 2',4,4'-Trihydroxychalcone | C_15_H_12_O_4_ | AM | Roots | NMRS; spectroscopy | Li, Zhou, Qiao, Fu, & Pei, 2007; Wang et al., 2014 |
| F46 | 2'-Methoxyisoliquiritigenin | C_16_H_14_O_4_ | AM | Roots | NMRS | Li, Li, & Yan, 2017 |
| F47 | 2',5'-Dicarbonyl-3',4'-dimethoxyisoflavanequinone 7-*O*-*β*-*D*-glucoside | C_23_H_26_O_11_ | AM | Roots | HPLC-ELSD | Wang, Han, Dai, Wang, & Ao, 2014 |
| F48 | 3'-Hydroxy-2,4-dimethoxyisoflavan 6-*O*-*β*-*D* -glucopyranoside | C_23_H_28_O_11_ | AM | Roots | HPLC-ELSD | Wang, Han, Dai, Wang, & Ao, 2014 |
| F49 | Ammopiptanoside A | C_26_H_26_O_10_ | AM | Roots | HPLC-ELSD | Zhang, Liu, Hsiao, Kuo, & Lee, 2011 |
| F50 | 4',7-Dihydroxy-3'-methoxy isoflavone | C_16_H_12_O_5_ | AM | Leaves | Spectroscopy | Wang, Zhu, Chen, Yu, & Ma, 2017; Wang et al., 2015 |
| F51 | (-)-Methylnissolin | C_17_H_16_O_5_ | AM | Roots | HPLC-ELSD | Lee et al., 2008; Zhang, Liu, Hsiao, Kuo, & Lee, 2011 |
| F52 | (-)-Methylinissolin 3-*O*-*β*-*D*-glucoside | C_23_H_26_O_10_ | AM | Roots | HPLC-ELSD | Zhang, Liu, Hsiao, Kuo, & Lee, 2011 |
| F53 | (-)-Methylinissolin 3-*O*-*β*-*D*-(6'-acetyl)-glucoside | C_25_H_28_O_11_ | AM | Roots | HPLC-ELSD | Zhang, Liu, Hsiao, Kuo, & Lee, 2011 |
| F54 | 8,3-Dihydroxy-7,4'-dimethoxy isoflavone | C_17_H_14_O_6_ | AM | Roots | Chemical and spectroscopic analysis | Song, Zh, Liu, & Hu, 1997 |
| F55 | Calycosin 7-*O*-*β*-*D*-{6''-[(E)-but-2-enoyl]}-glucopyranoside | C_17_H_14_O_6_ | AMM | Roots | HPLC-ELSD | Pei et al., 2007; Zhang, Liu, Hsiao, Kuo, & Lee, 2011 |
| F56 | 7,3'-dihydroxy-8,4'-dimethoxy isoflavone | C_26_H_26_O_11_ | AM | Roots | Chemical and spectroscopic analysis | Song, Zh, Liu, & Hu, 1997 |
| F57 | Liquiritigenin | C_15_H_12_O_4_ | AM | Roots | NMRS | Li, Li, & Yan, 2017 |
| F58 | Pratensein | C_16_H_12_O_6_ | AM | Roots | NMRS | Li, Li, & Yan, 2017 |
| F59 | Odoratin 7‐*O*‐*β*‐*D*‐glucopyranoside | C_23_H_24_O_11_ | AM | Roots; steams | Chemical and spectroscopic analysis | Bi, Yu, Li, Lin, & Gao, 2007; Hao, Li, Li, Liu, & Ruan, 2016 |
| F60 | (-)-Methylinissolin 3-*O*-*β*-*D*-{6'‐*O*‐[(E)‐but‐2‐enoyl]}-glucoside | C_27_H_30_O_11_ | AM | Roots | HPLC-ELSD | Tu et al., 2009; Zhang, Liu, Hsiao, Kuo, & Lee, 2011 |
| F61 | Vesticarpan | C_16_H_14_O_5_ | AM | Roots | HPLC-ELSD | Zhang, Liu, Hsiao, Kuo, & Lee, 2011 |
| F62 | Licoagroside D | C_22_H_24_O_10_ | AM | Roots | HPLC-ELSD | Zhang, Liu, Hsiao, Kuo, & Lee, 2011 |
| F63 | Trifolinhizin | C_22_H_22_O_10_ | AM | Roots | Spectroscopic analysis | Wang, Han, Dai, Wang, & Ao, 2014 |
| F64 | Astraflavonoid C | C_23_H_28_O_11_ | AM | Stems | Chemical and spectroscopic analysis | Hao, Li, Li, Liu, & Ruan, 2016 |
| F65 | Echinatin | C_16_H_14_O_4_ | AM | Roots | NMRS | Li, Li, & Yan, 2017 |
| F66 | Licochalcone B | C_16_H_14_O_5_ | AM | Roots | NMRS | Li, Li, & Yan, 2017 |
| F67 | (3*R*,4*R*)-4,7-Hydroxy-2',3'-dimethoxyisoflavane 4'-*O*-*β*-*D*-glucoside | C_23_H_28_O_11_ | AM | Roots | Spectroscopic analysis | Wang et al., 2014 |
| F67 | Sophorophenolone | C_16_H_10_O_6_ | AM | Roots | HPLC-ELSD | Kim, Yean, Lee, Jung, & Lee, 2008 |
| F68 | Adenosine | C_10_H_13_N_5_O_4_ | AM | Roots | NMRS | Kim, Yean, Lee, Jung, & Lee, 2008 |
| F69 | Astramemoside A | C_18_H_22_O_11_ | AM | Stems | Chemical and spectroscopic analysis | Hao, Li, Li, Liu, & Ruan, 2016 |
| F70 | Emodin | C_15_H_10_O_5_ | AM | − | Spectroscopy | Zheng & Wang, 2011 |
| F71 | Isorhamnetin-3-*O*-[*β*-*D*-glucopyranosyl-(1→5)-*β*-*D*-apiofuranosyl] - (1→2)-robinobioside | C_39_H_50_O_25_ | AM | Aerial parts | LC-MS; NMRS | Vasilev et al., 2019 |
| F72 | Isorhamnetin-3-*O*-(2,6-di-*O*-*α*-rhamnopyranosyl-*β*-*D*-galactopyranoside)-7-*O*-*β*-*D*-glucopyranoside | C_40_H_52_O_25_ | AM | Aerial parts | LC-MS; NMRS | Vasilev, Ross, Smejkal, Marsík, & Jankovská, 2019 |
| F73 | Isorhamnetin-3-*O*-robinobioside-7-*O*-glucoside | C_34_H_42_O_21_ | AM | Aerial parts | LC-MS; NMRS | Vasilev, Ross, Smejkal, Marsík, & Jankovská, 2019 |
| F74 | Astragaisoflavan B | C_17_H_18_O_6_ | AM | Aerial parts | Spectroscopy | Guo et al., 2016 |
| F75 | Astragaisoflavan A | C_17_H_18_O_6_ | AM | Aerial parts | Spectroscopy | Guo, He, Zhang, Li, & Yan, 2016 |
| F76 | Astragaisoflavan C | C_18_H_24_O_7_ | AM | Aerial parts | Spectroscopy | Guo, He, Zhang, Li, & Yan, 2016 |
| F77 | Astragaisoflavan *D* | C_34_H_34_O_10_ | AM | Aerial parts | Spectroscopy | Guo, He, Zhang, Li, & Yan, 2016 |
| F78 | 4‐Hydroxycinnamic acid | C_9_H_8_O_3_ | AM | Roots | NMRS | Li, Li, & Yan, 2017 |
| F79 | Gentisin | C_14_H_10_O_5_ | AM | − | Spectroscopy | Zheng & Wang, 2011 |

**Table S2** Chemical constituents in Astragaloside compounds.

| No. | Names | Molecular formula | Species | Parts | Appraisal | References |
| --- | --- | --- | --- | --- | --- | --- |
| S1 | 3-*O*-*β*-*D*-Xylopyranosyl-6*O*-*β*-*D*-glucopyranosyl-3*β*,6*α*,16*β*,24(*S*)-25-Pentahydroxycycloartane dodecaacetate | C_73_H_106_O_32_ | AM | Roots | ESIMS, HRMS | Un et al., 2016 |
| S2 | 6*α*,16*β*,24(*S*),25-Tetrahydroxycycloartane-3-one | C_30_H_50_O_5_ | AM | − | NMR, ESIMS, HRMS | Aslanipour, Gülcemal, Nalbantsoy, Yusufoglu, & Bedir, 2017 |
| S3 | 6-*O*-*β*-*D*-Xylopyranosyl-3*β*,6*α*,16*β*,24(*S*),25-pentahydroxycycloartane | C_35_H_60_O_9_ | AM | − | NMR, ESIMS, HRMS | Aslanipour, Gülcemal, Nalbantsoy, Yusufoglu, & Bedir, 2017 |
| S4 | Huangqiyenin A | C_36_H_58_O_10_ | AM | Stems | Chemical and spectroscopic analysis | Wang, Ruan, Li, Chao, & Shi, 2016 |
| S5 | Huangqiyegenin I | C_30_H_48_O_5_ | AM | Leaves | Spectral analysis | Wang, Zhai, Ma, Yang, & Pan, 2015 |
| S6 | Huangqiyegenin V | C_30_H_46_O_6_ | AM | Leaves | Spectral analysis | Wang, Zhai, Ma, Yang, & Pan, 2015 |
| S7 | Isoastragaloside IV | C_41_H_68_O_14_ | AM | Roots | Chemical and spectroscopic analysis | Li et al., 2017; Wang, Tang, & Duan, 2017 |
| S8 | Astragaloside VII | C_47_H_78_O_19_ | AM | Roots | Chemical and spectroscopic analysis | Hao, Li, Li, Liu, & Ruan, 2016; Zhou et al., 2016 |
| S9 | Astragaloside II | C_43_H_70_O_15_ | AM | Roots | NMR | Lee et al., 2019; Wen, Cheng, Zheng, Huang, & Han, 2010 |
| S10 | Isoastragaloside I | C_45_H_72_O_16_ | AM | Roots | Spectral analysis | Li, Li, Yan, & Su, 2017; Zhang, Liu, Hsiao, Kuo, & Lee, 2011 |
| S11 | Acetylastragaloside I | C_47_H_74_O_17_ | AM | Roots | Chemical and spectroscopic analysis | Bian, Guan, Bi, Song, & Li, 2006; Hao, Li, Li, Liu, & Ruan, 2016; Lee et al., 2019 |
| S12 | Isoastragaloside II | C_43_H_70_O_15_ | AM | Roots | HR-FAB/MS | Bian, Guan, Bi, Song, & Li, 2006; Kim et al., 2022; Li, Li, Yan, & Su, 2017; Zhang, Liu, Hsiao, Kuo, & Lee, 2011 |
| S13 | Astragaloside I | C_45_H_72_O_16_ | AM | Roots | Chemical and spectroscopic analysis | Cao et al., 1985; Hao, Li, Li, Liu, & Ruan, 2016; Li, Li, Yan, & Su, 2017; Zhang, Liu, Hsiao, Kuo, & Lee, 2011 |
| S14 | Astramembranoside A | C_42_H_70_O_15_ | AM | Roots | Chemical and spectroscopic analysis | Kim et al., 2008 |
| S15 | Brachyoside B | C_36_H_60_O_10_ | AM | Roots | Chemical and spectroscopic analysis; NMRS | Kim et al., 2008; Li, Li, Yan, & Su, 2017 |
| S16 | Astragaloside VI | C_47_H_78_O_19_ | AM | Roots | Spectral analysis | Wang, Tang, & Duan, 2017 |
| S17 | Astragaloside IV | C_41_H_68_O_14_ | AM | Roots; leaves | Spectral analysis | Bian, Guan, Bi, Song, & Li, 2006; Li, Li, Yan, & Su, 2017; Zhang, Liu, Hsiao, Kuo, & Lee, 2011 |
| S18 | Astragaloside V | C_47_H_78_O_19_ | AM | Roots | Spectral analysis | Wang, Tang, & Duan, 2017 |
| S19 | Cycloaraloside A | C_36_H_60_O_10_ | AM | Roots; leaves | Chemical and spectroscopic analysis; NMRS | Li, Li, Yan, & Su, 2017; Wang, Ruan, Li, Chao, & Shi, 2016 |
| S20 | Astramembrannin II | C_35_H_58_O_9_ | AM | Roots | Spectrum analysis | Bian, Guan, Bi, Song, & Li, 2006; Cao, Yu, Gan, & Chen, 1985 |
| S21 | Astraverrucin II | C_38_H_62_O_11_ | AM | Stems | Chemical and spectroscopic analysis | Wang, Ruan, Li, Chao, & Shi, 2016 |
| S22 | Astrolanosaponin A_1_ | C_42_H_70_O_15_ | AM | Stems | Chemical and spectroscopic analysis | Bian, Guang, Bi, Song, & Li, 2006; Wang et al., 2016 |
| S23 | Cycloaraloside E | C_42_H_70_O_15_ | AM | Stems | Chemical and spectroscopic analysis | Wang, Ruan, Li, Chao, & Shi, 2016 |
| S24 | Astrolanosaponin A_2_ | C_44_H_72_O_16_ | AM | Stems | Chemical and spectroscopic analysis | Wang, Ruan, Li, Chao, & Shi, 2016 |
| S25 | Cyclocanthoside A | C_35_H_60_O_9_ | AM | Roots | Spectral analysis | Wang, Tang, & Duan, 2017 |
| S26 | Astramembranoside B | C_41_H_70_O_14_ | AM | Roots | Spectral analysis | Kim, Yean, Lee, Jung, & Lee, 2008; Wang, Zhai, Ma, Yang, & Pan, 2015 |
| S27 | Cyclocanthoside E | C_41_H_70_O_14_ | AM | Roots | Spectral analysis; NRMS | Li, Li, Yan, & Su, 2017 |
| S28 | Agroastragaloside II | C_43_H_72_O_15_ | AM | Roots | HR-FAB/MS | Hirotani et al., 1994; Kim et al., 2013 |
| S29 | Agroastragaloside I | C_45_H_74_O_16_ | AM | Roots | HR-FAB/MS | Lee et al., 2013 |
| S30 | Azukisaponin V | C_48_H_78_O_18_ | AM | Leaves | Spectral analysis | Zhang et al., 2016; Wang, Zhai, Ma, Yang, & Pan, 2015 |
| S31 | Soyasaponin I | C_48_H_78_O_18_ | AM | Roots | Spectral analysis | Zheng & Wang, 2011 |
| S32 | Mongholicoside I | C_36_H_60_O_9_ | AM | Aerial parts | Chemical and spectroscopic analysis | Zhu et al., 1992 |
| S33 | Mongholicoside A | C_36_H_62_O_11_ | AM | Aerial parts | NMR, ESI-MS | Yu et al., 2007 |
| S34 | Mongholicoside B | C_36_H_60_O_11_ | AM | Aerial parts | NMR, ESI-MS | Yu, Li, Bi, Luo, & Gao, 2007 |
| S35 | Huangqiyenin L | C_43_H_70_O_14_ | AM | Leaves | Spectral analysis | Wang, Zhai, Ma, Yang, & Pan, 2015; Zhang, Liu, Hsiao, Kuo, & Lee, 2011 |
| S36 | Astroolesaponin A | C_48_H_76_O_18_ | AM | Stems | Chemical and spectroscopic analysis | Zhang et al., 2016 |
| S37 | Cloversaponin III | C_42_H_64_O_16_ | AM | Stems | Chemical and spectroscopic analysis | Zhang, Li, Ruan, Wang, & Dong, 2016 |
| S38 | Astroolesaponin E_1_ | C_42_H_64_O_17_ | AM | Stems | Chemical and spectroscopic analysis | Zhang, Li, Ruan, Wang, & Dong, 2016 |
| S39 | Astroolesaponin E_2_ | C_41_H_62_O_16_ | AM | Stems | Chemical and spectroscopic analysis | Zhang, Li, Ruan, Wang, & Dong, 2016 |
| S40 | Astraisoolesaponin B | C_42_H_62_O_16_ | AM | Stems | Chemical and spectroscopic analysis | Zhang, Li, Ruan, Wang, & Dong, 2016 |
| S41 | Astraisoolesaponin A_2_ | C_42_H_62_O_17_ | AM | Stems | Chemical and spectroscopic analysis | Zhang, Li, Ruan, Wang, & Dong, 2016 |
| S42 | Astraisoolesaponin A_3_ | C_41_H_60_O_16_ | AM | Stems | Chemical and spectroscopic analysis | Zhang, Li, Ruan, Wang, & Dong, 2016 |
| S43 | Astraisoolesaponin A_1_ | C_48_H_72_O_21_ | AM | Stems | Chemical and spectroscopic analysis | Zhang, Li, Ruan, Wang, & Dong, 2016 |
| S44 | Astroolesaponin F | C_49_H_78_O_18_ | AM | Stems | Chemical and spectroscopic analysis | Zhang, Li, Ruan, Wang, & Dong, 2016 |
| S45 | Astroolesaponin C_1_ | C_43_H_68_O_16_ | AM | Stems | Chemical and spectroscopic analysis | Zhang, Li, Ruan, Wang, & Dong, 2016 |
| S46 | Robinioside B | C_48_H_76_O_20_ | AM | Stems | Chemical and spectroscopic analysis | Zhang, Li, Ruan, Wang, & Dong, 2016; Zhang, Li, Ruan, Wang, & Dong, 2016 |
| S47 | Astroolesaponin C_2_ | C_49_H_78_O_20_ | AM | Stems | Chemical and spectroscopic analysis | Zhang, Li, Ruan, Wang, & Dong, 2016 |
| S48 | Robinioside F | C_48_H_78_O_19_ | AM | Stems | Chemical and spectroscopic analysis | Zhang, Li, Ruan, Wang, & Dong, 2016 |
| S49 | Astroolesaponin *D* | C_48_H_74_O_19_ | AM | Stems | Chemical and spectroscopic analysis | Zhang, Li, Ruan, Wang, & Dong, 2016 |
| S50 | Astroolesaponin B | C_48_H_78_O_19_ | AM | Stems | Chemical and spectroscopic analysis | Zhang, Li, Ruan, Wang, & Dong, 2016 |
| S51 | Astrolanosaponin B | C_42_H_68_O_15_ | AM | Stems | Chemical and spectroscopic analysis | Wang, Ruan, Li, Chao, & Shi, 2016; Zhang, Li, Ruan, Wang, & Dong, 2016 |
| S52 | Astrolanosaponin E | C_36_H_60_O_11_ | AM | Stems | Spectroscopy | Lee, Lee, Choi, Lee, & Jang, 2019; Wang, Ruan, Li, Chao, & Shi, 2016; Wen, Cheng, Zheng, Huang, & Han, 2010 |
| S53 | Lupeol | C_30_H_50_O | AM | Roots | MS | He & Wang, 1990 |
| S54 | Ursolic acid | C_30_H_48_O_3_ | AM | Roots | Spectroscopy | Zheng & Wang, 2011 |
| S55 | Soyasapogenol B | C_30_H_50_O_3_ | AM | Leaves | Spectroscopy | Zhang, Liu, Hsiao, Kuo, & Lee, 2011 |
| S56 | Soyasapogenol E | C_30_H_48_O_3_ | AM | Leaves | Spectroscopy | Zhang, Liu, Hsiao, Kuo, & Lee, 2011 |
| S57 | Huangqiyenin F | C_40_H_64_O_12_ | AM | Leaves | NMR | Kuang et al., 2009 |
| S58 | Huangqiyenin G | C_40_H_62_O_13_ | AM | Leaves | NMR | Kuang et al., 2011 |
| S59 | Huangqiyenin E | C_42_H_66_O_14_ | AM | Leaves; stems | NMR | Kuang, Okada, Yang, Tian, & Okuyama, 2009; Wang, Ruan, Li, Chao, & Shi, 2016 |
| S60 | Huangqiyenin H | C_40_H_60_O_13_ | AM | Leaves | NMR | Kuang, Wang, Yang, Wang, & Okada, 2011 |
| S61 | Huangqiyenin J | C_40_H_62_O_12_ | AM | Leaves | NMR | Kuang, Wang, Yang, Wang, & Okada, 2011 |
| S62 | Huangqiyenin I | C_40_H_64_O_13_ | AM | Leaves | NMR | Kuang, Wang, Yang, Wang, & Okada, 2011 |
| S63 | Huangqiyenin K | C_37_H_60_O_10_ | AM | Leaves | NMR | Kuang, Wang, Yang, Wang, & Okada, 2011; Wang, Zhai, Ma, Yang, & Pan, 2015 |
| S64 | Huangqiyenin B | C_36_H_60_O_10_ | AM | Stems | Chemical and spectroscopic analysis | Wang, Ruan, Li, Chao, & Shi, 2016 |
| S65 | Mongholicoside II | C_38_H_62_O_11_ | AM | Aerial part | Spectroscopy | Zhu, Lu, Okada, Takata, & Okuyama, 1992 |
| S66 | 29-*O*-*α*-*L*-rhamnopyranosyl-abrisapogenol B | C_36_H_60_O_8_ | AM | Roots | ESIMS, HRMS | Un, Horo, Masullo, Falco, & Senol, 2016 |
| S67 | Astroolesaponin A | C_48_H_76_O_18_ | AM | Stems | Chemical and spectroscopic analysis | Zhang, Li, Ruan, Wang, & Dong, 2016 |
| S68 | Astroolesaponin B | C_48_H_78_O_19_ | AM | Stems | Chemical and spectroscopic analysis | Zhang, Li, Ruan, Wang, & Dong, 2016 |
| S69 | Astroolesaponin E_1_ | C_42_H_64_O_17_ | AM | Stems | Chemical and spectroscopic analysis | Zhang, Li, Ruan, Wang, & Dong, 2016 |
| S70 | Astroolesaponin E_2_ | C_41_H_62_O_16_ | AM | Stems | Chemical and spectroscopic analysis | Zhang, Li, Ruan, Wang, & Dong, 2016 |
| S71 | Astroolesaponin *D* | C_48_H_74_O_19_ | AM | Stems | Chemical and spectroscopic analysis | Zhang, Li, Ruan, Wang, & Dong, 2016 |
| S73 | Astragaloside III | C_41_H_68_O_14_ | AM | Roots | Spectral analysis; NRMS | Li, Li, Yan, & Su, 2017; Zhang, Liu, Hsiao, Kuo, & Lee, 2011 |

**Table S3** Characteristics of *Astragalus* polysaccharides (APS).

| NO． | Names | Monosaccharide composition | Molecular weight | Molar ratio | References |
| --- | --- | --- | --- | --- | --- |
| A1 | APS-A1 | 1,4,6-*α*-*D*-Glcp | 2.62 × 10^6^ | − | Chen et al., 2023 |
| A2 | APS-B1 | Glucose, galactose and arabinose | 4.95 × 10^6^ | 75.24:17.27:19.35 | Chen, Jiang, Zheng, Hu, & Yang, 2023 |
| A3 | APS2-I | Man, Rha, GlcA, GalA, Glc, Gal, Xyl, and Ara | 1 .96 × 10^6^ | 2.3:4.8:1.7:14.0:5.8:11.7:2.8:12.6 | Wang et al., 2023 |
| A4 | APS3-I | Rha, GalA, Glc, Gal, and Ara | 3.91 × 10^6^ | 0.8:2.3:0.8:2.3:4.1 | Wang, Peng, Zhuang, Wang, & Jin, 2023 |
| A5 | APS-I | *D*-Galactose, *D*-Glucose | 3.84 × 10^4^ | 1:49.76 | Wang, Peng, Zhuang, Wang, & Jin, 2023 |
| A6 | APS-I | *L*-Rhamnose, *D*-Galacturonic acid, *D*-Galactose, *D*-Glucose, *L*-Arabinose | 2 × 10^5^ | 0.1:0.39:13.4:17.2:1 | Wang, Peng, Zhuang, Wang, & Jin, 2023 |
| A7 | APS-II | *L*-Rhamnose, *D*-Galacturonic acid, *D*-Galactose, *D*-Glucose, *L*-Arabinose | 1 × 10^4^ | 0.14:0.14:9.6:24.04:1 | Wang, Peng, Zhuang, Wang, & Jin, 2023 |
| A8 | APS-II | *L*-Rhamnose, *D*-Galactose, *D*-Glucose | 5.2 × 10^3^ | 1:2.99:16.26 | Wang, Peng, Zhuang, Wang, & Jin, 2023 |
| A9 | APS-III | *L*-Rhamnose, *D*-Galacturonic acid, *D*-Galactose, *D*-Glucose, *L*-Arabinose | 3.0 × 10^2^ | 0.375:0.375:18.8:90.5:1 | Wang, Peng, Zhuang, Wang, & Jin, 2023 |
| A10 | APS-1 | *D*-Glucose | 2.57 × 10^5^ | − | Jiang et al., 2016 |
| A11 | APS-2 | *L*-Arabinose | 4.01 × 10^4^ | − | Jiang, Qi, Gao, Liu, & Li, 2016 |
| A12 | APS-3 | *L*-Rhamnose, *D*-Glucose, *D*-Galactose, *L*-Arabinose | 1.53 × 10^4^ | 1:10.76:6.55:12 | Jiang, Qi, Gao, Liu, & Li, 2016 |
| A13 | APS-4 | *D*-Galactose, *L*-Arabinose | 3.2 × 10^3^ | 3.02:1 | Jiang, Qi, Gao, Liu, & Li, 2016 |
| A14 | LM_W_-ASP | Glc, Gal, Ara, Xyl, and GalA | 5.6 × 10^3^ | 10.0:1.3:1.7:1.0:0.9 | Jiang, Qi, Gao, Liu, & Li, 2016 |
| A15 | APS-I | *D*-Mannose, *L*-Rhamnose, *D*-Glucuronic acid, D-Galacturonic | 1.06 × 10^4^ | 29.12:1.89:4.00:1.35:1:81.97 | Fu et al., 2013 |
| A16 | APS-II | *D*-Mannose, *L*-Rhamnose, *D*-Glucuronic acid, *D*-Galacturonic  acid, *D*-Glucose, *D*-Galactose, *D*-Xylose | 2.47 × 10^6^ | 50.46:1.16:1:2.27:2.66:15.72:7.86 | Fu, Huang, Zhang, Yang & Chen, 2013 |
| A17 | ASP-I | *D*-Galactose, *D*-Glucose, *L*-Arabinose | 4.32 × 10^4^ | 1.0:24.8:2.5 | Wang et al., 2023 |
| A18 | ASP-II | *L*-Rhamnose, *D*-Galacturonic acid, *D*-Glucose, *D*-Galactose, | 2.81 × 10^5^ | 1.2:1.0:19.3:2.5:8.7 | Wang, Wang, Zhang, Cao, & Kong, 2023 |
| A19 | MAPS-5 | *α*-*D*-(1–4) Glucos | 1.32 × 10^4^ |  | Sheng et al., 2025 |
| A20 | APS | *D*-Mannose, *D*-Glucose, *D*-Xylose, *L*-Arabinose, *D*-Glucuronic | 2 × 10^6^ | 0.27:12.83:1.63:0.71:1.04:0.56 | Wu et al., 2021 |

**Table S4** Chemical structure of *Astragalus* flavonoids.

| No. | Names | Parent nucleus | Substituents |
| --- | --- | --- | --- |
| **F1** | Quercetin |  | R=H |
| **F2** | Quercetin 3‐*O*‐*β*‐*D*‐glucopyranoside |  | R=Glc |
| **F3** | Astraflavonoid A |  | R_1_=5-*O*-[(*E*)-*p*-feruloyl]-Api(f)-(1→2)-Glc,  R_2_=H |
| **F4** | Kaempferol |  | R_1_=H,  R_2_=H |
| **F5** | Kaempferol 3‐*O*‐*β*‐*D*‐glucoside |  | R_1_=Glc,  R_2_=H |
| **F6** | Kaempferol 3‐*O*‐(2‐*O*‐*α*‐*L*‐rhamnopyranosyl)‐*β*‐*D*‐glucopyranoside |  | R_1_=Rha-(1→2)-Glc,  R_2_=H |
| **F7** | Rhamnocitrin 3‐*O*‐neohesperoside |  | R_1_=Rha-(1→2)-Glc,  R_2_=Me |
| **F8** | Kaempferol 3,7‐di‐*O*‐*β*‐*D*‐glucopyranoside |  | R_1_=Glc,  R_2_=Glc |
| **F9** | Astraflavonoid B |  | R_1_=6-*O*-[(*E*)-*p*-feruloyl]-Glc,  R_2_=Rha-(1→2)-Glc |
| **F10** | Isorhamnetin |  | R_1_=H, R_2_=H, R_3_=Me |
| **F11** | Quercetin3‐*O*‐*β*‐*D*‐neospheroside (Rutin) |  | R_1_=Rha-(1→2)-Glc, R_2_=H, R_3_=OH |
| **F12** | Isorhamnetin3‐*O*‐*β*‐*D*‐glucoside |  | R_1_=Glc, R_2_=H, R_3_=Me |
| **F13** | Kaempferol 4'‐methoxyl‐3‐*O*‐*β*‐*D*‐glucoside |  | R_1_=Glc, R_2_=CH_3_, R_3_=H |
| **F14** | Oroxylin-A |  | R_1_=H, R_2_=OMe |
| **F15** | Wogonin |  | R_1_=OMe, R_2_=H |
| **F16** | 4',7-*D*ihydroxyflavone |  | R=H |
| **F17** | 3',4',7‐Trihydroxy flavone |  | R=OH |
| **F18** | Rhamnocitrin 3‐*O*‐*β*‐*D*‐glucopyranoside |  | R_1_=Glc, R_2_=H |
| **F19** | Complanatuside |  | R_1_=Glc, R_2_=Glc |
| **F20** | Rhamnocitrin 3‐*O*‐*β*‐*D*‐glucopyranoside (1 → 2)‐*β*‐*D*‐apiofuranosyl |  | R_1_=Api(f)-(1→2)-Glc, R_2_=H |
| **F21** | Rhamnocitrin 3‐*O*‐*β*‐neohesperidoside |  | R_1_=Rha-(1→2)-Glc, R_2_=H |
| **F22** | Tiliroside |  | R_1_=6-*O*-[(*E*)-*p*-coumaroyl]-Glc, R_2_=H |
| **F23** | Isorhamnetin 3‐*O*‐(6‐*O*‐*α*‐*L*‐fucopyranosyl) ‐*β*‐*D*‐glucopyranoside |  | R_1_=Fuco-(1→6)-Glc, R_2_=OMe |
| **F24** | 6''‐Acetyl‐ononin |  | R_1_=H, R_2_=H |
| **F25** | Formononetin |  | R_1_=Glc, R_2_=H |
| **F26** | Formononetin 7‐*O*‐*β*‐*D*‐glucoside (Ononin) |  | R_1_=6-*O*-Ac-Glc, R_2_=H |
| **F27** | Calycosin |  | R=H, |
| **F28** | Calycosin 7‐*O*‐*β*‐*D*‐glucopyranoside |  | R=Glc |
| **F29** | Genistein |  | R=H |
| **F30** | Genistin |  | R=Glc |
| **F31** | Glycitein |  | R=H |
| **F32** | Glycitin |  | R=Glc |
| **F33** | 3',7-Dihydroxy-5'-methoxyisoflavone |  | − |
| **F34** | 4',5,7-Trihydroxy-3'-methoxyisoflavone |  | R_1_=H, R_2_=OCH_3_, R_3_=H |
| **F35** | Pratensein 7-*O*-*β*-*D*-glucopyranoside |  | R_1_=OGlc, R_2_=H, R_3_=Me |
| **F36** | Sissotrin |  | R_1_=H, R_2_=H, R_3_=Rha-(1→2)-Glc |
| **F37** | Sophorabioside |  | R_1_=H, R_2_=H, R_3_=Rha-(1→2)-Glc |
| **F38** | Pendulone |  | − |
| **F39** | (3*R*)-8,2'-Dihydroxy-7,4'-dimethoxyisoflavan |  | − |
| **F40** | Isomucronulatol |  | R_1_=H, R_2_=H, R_3_=OCH_3_ |
| **F41** | Isomucronulatol-7-*β*-*O*-glucoside |  | R_1_=OH, R_2_=Glc, R_3_=OCH_3_ |
| **F42** | 4,4',6'-Trihydroxychalcone |  | R=H |
| **F43** | 4-Methoxy-4',6'-dihydroxychalcone |  | R=Me |
| **F44** | 4,4'-*D*imethyl-6'-hydroxy chalcone |  | − |
| **F45** | 2',4,4'-Trihydroxychalcone |  | R_1_=H, R_2_=H |
| **F46** | 2'-Methoxyisoliquiritigenin |  | R_1_=OH, R_2_=Me |
| **F47** | 2',5'-*D*icarbonyl-3',4'-dimethoxyisoflavanequinone 7-*O*-*β*-*D*-glucoside |  | − |
| **F48** | 3'-Hydroxy-24-dimethoxyisoflavan 6-*O*-*β*-*D*-glucopyranoside |  | − |
| **F49** | Ammopiptanoside A |  | R_1_=6-*O*-[(*E*)-but-2-enoyl]-Glc, R_2_=CH_3_, R_3_=H |
| **F50** | 4',7-Dihydroxy-3'-methoxy isoflavone |  | R_1_=H, R_2_=H, R_3_=CH_3_ |
| **F51** | (-)-Methylnissolin |  | R=H |
| **F52** | (-)-Methylinissolin 3-*O*-*β*-*D*-glucoside |  | R=Glc |
| **F53** | (-)-Methylinissolin 3-*O*-*β*-*D*-(6'-acetyl)-glucoside |  | R=6-O-Ac-Glc |
| **F54** | 8,3-Dihydroxy-7,4'-dimethoxy isoflavone |  | R_1_=OH, R_2_=OMe |
| **F55** | Calycosin 7-*O*-*β*-*D*-{6''-[(*E*)-but-2-enoyl]}-glucopyranoside |  | R_1_=H, R_2_=6-*O*-[(*E*)-but-2-enoyl]-Glc |
| **F56** | 7,3'-Dihydroxy-8,4'-dimethoxy isoflavone |  | R_1_=OMe, R_2_=H |
| **F57** | Liquiritigenin |  | − |
| **F58** | Pratensein |  | R_1_=H, R_2_=OH, R_3_=OH |
| **F59** | Odoratin 7‐*O*‐*β*‐*D*‐glucopyranoside |  | R_1_=H, R_2_=OMe, R_3_=Glc |
| **F60** | (-)-Methylinissolin 3-*O*-*β*-*D*-{6'‐*O*‐[(E)‐but‐2‐enoyl]}-glucoside |  | R_1_=H, R_2_=H |
| **F61** | Vesticarpan |  | R_1_=Glc, R_2_=H |
| **F62** | Licoagroside *D* |  | R_1_=6-*O*-[(E)-but-2-enoyl]-Glc, R_2_= Me |
| **F63** | Trifolinhizin |  | − |
| **F64** | Astraflavonoid C |  | − |
| **F65** | Echinatin |  | R=H |
| **F66** | Licochalcone B |  | R=OH |
| **F67** | Sophorophenolone |  | − |
| **F68** | Adenosine |  | − |
| **F69** | Astramemoside A |  | − |
| **F70** | Emodin |  | − |
| **F71** | Isorhamnetin-3-*O*-[*β*-*D*-glucopyranosyl-(1→5)-*β*-*D*-apiofuranosyl]-(1→2)-robinobioside |  | R_1_=H, R_2_=OMe, R_3_=*O*-[*β*-*L*-api-(1→2)-*α*-*L*-Rha-(1→6)]-*β*-*D*-Gal], R_4_=*β*-*D*-Glc |
| **F72** | Isorhamnetin-3-*O*-(2,6-di-*O*-*α*-rhamnopyranosyl-*β*-*D*-galactopyranoside)-7-*O*-*β*-*D*-glucopyranoside |  | R_1_=H, R_2_=OMe, R_3_=*O*-[*β*-*D*-Glc(1→5)-*β*-*D*-api-[*α*-*L*-Rha(1→6)]-*β*-*D*-Gal], R_4_=H |
| **F73** | Isorhamnetin-3-*O*-robinobioside-7-*O*-glucoside |  | R_1_=H, R_2_=OMe, R_3_=*O*-(2,6-di-*O*-*α*-Rha-*β*-*D*-Gal), R_4_=*β*-*D*-Glc |
| **F74** | Astragaisoflavan B |  | − |
| **F75** | Astragaisoflavan A |  | − |
| **F76** | Astragaisoflavan C |  | − |
| **F77** | Astragaisoflavan D |  | − |
| **F78** | 4‐Hydroxycinnamic acid |  | − |
| **F79** | Gentisin |  | − |

**Table S5** Chemmical structure of *Astragalus* saponins.

| No. | Names | Parent nucleus | Substituents |
| --- | --- | --- | --- |
| **S1** | 3-*O*-*β*-*D*-xylopyranosyl-6,16-di-*O*-*β*-*D*-glucopyranosyl-3*β*,6*α*,16*β*,24(*S*)-25-pentahydroxycycloartane dodecaacetate |  | R_1_=CH_3_CHCH_2_CH_2_CH(OH)C(CH_3_)_2_OH, R_2_=*O*-*β*-*D*-Glc, R_3_=*O*-*β*-*D*-Glc, R_4_=*α*-*L*-Ara-(1→2)-*β*-*D*-Xyl |
| **S2** | 6*α*,16*β*,24(*S*),25-tetrahydroxycycloartane-3-one |  | R_1_=CH_3_CHCH_2_CH_2_CH(OH)C(CH_3_)_2_OH, R_2_=OH, R_3_=OH, R_4_= O |
| **S3** | 6-*O*-*β*-*D*-xylopyranosyl-3*β*,6*α*,16*β*,24(*S*),25-pentahydroxycycloartane |  | R_1_=CH_3_CHCH_2_CH_2_CH(OH)C(CH_3_)_2_OH, R_2_=OH, R_3_=*O*-*β*-*D*-Xyl, R_4_=OH |
| **S4** | Huangqiyenin A |  | − |
| **S5** | Huangqiyegenin I |  | − |
| **S6** | Huangqiyegenin V |  | − |
| **S7** | Isoastragaloside IV |  | R=H |
| **S8** | Astragaloside VII |  | R=Glc |
| **S9** | Astragaloside II |  | R=2-*O*-Ac-Xyl |
| **S10** | Isoastragaloside I |  | R=2,4-*O*-Ac_2_-Xyl |
| **S11** | Acetylastragaloside I |  | R=2,3,4-*O*-Ac_3_-Xyl |
| **S12** | Isoastragaloside II |  | R=3-*O*-Ac-Xyl |
| **S13** | Astragaloside I |  | R=2,3-*O*-Ac_2_-Xyl |
| **S14** | Astramembranoside A |  | R=H |
| **S15** | Brachyoside B |  | R=H |
| **S16** | Astragaloside IV |  | R=XylO |
| **S17** | Astragaloside VI |  | R=Glc-(1→2)-Xyl, R_1_=Glc, R_2_=H |
| **S18** | Astragaloside V |  | R=Glc-(1→2)-Xyl, R_1_=H, R_2_=H |
| **S19** | Cycloaraloside A |  | R=Glc |
| **S20** | Astramembrannin II |  | R=Xyl |
| **S21** | Astraverrucin II |  | R_1_=2-*O*-Ac-Glc, R_2_=R_3_=H |
| **S22** | Astrolanosaponin A_1_ |  | R_1_= R_2_= Glc, R_3_=H |
| **S23** | Cycloaraloside E |  | R_1_= R_3_= Glc, R_2_=H |
| **S24** | Astrolanosaponin A_2_ |  | R_1_=2-O-Ac-Glc, R_2_=H, R_3_= Glc |
| **S25** | Cyclocanthoside A |  | R_1_=Xyl, R_2_=H |
| **S26** | Astramembranoside B |  | R_1_=Glc-(1→2)-Xyl, R_2_=H |
| **S27** | Cyclocanthoside E |  | R_1_=Xyl, R_2_=Glc |
| **S28** | Agroastragaloside II |  | R_1_=2-O-Ac-Xyl, R_2_=Glc |
| **S29** | Agroastragaloside I |  | R_1_=2,3-*O*-AC_2_-Xyl, R_2_=Glc |
| **S30** | Azukisaponin V |  | R= Rha- (1→2) - Glc-(1→2)- GlcA |
| **S31** | Soyasaponin I |  | R=Rha-(1→2)-Gal-(1→2)-GlcA |
| **S32** | Mongholicoside I |  | − |
| **S33** | Mongholicoside A |  | R=β-OH |
| **S34** | Mongholicoside B |  | R= OH |
| **S35** | Huangqiyenin L |  | − |
| **S36** | Astroolesaponin A |  | R_1_=Rha-(1→2)-Glc-(1→2)-GlcA, R_2_=H |
| **S37** | Cloversaponin III |  | R_1_=Glc-(1→2)-GlcA, R_2_=H |
| **S38** | Astroolesaponin E_1_ |  | R_1_=Glc-(1→2)-GlcA, R_2_=OH |
| **S39** | Astroolesaponin E_2_ |  | R_1_=Xyl-(1→2)-GlcA, R_2_=OH |
| **S40** | Astraisoolesaponin B |  | R_1_=Glc-(1→2)-GlcA, R_2_=H |
| **S41** | Astraisoolesaponin A_2_ |  | R_1_=Glc-(1→2)-GlcA, R_2_=OH |
| **S42** | Astraisoolesaponin A_3_ |  | R_1_=Xyl-(1→2)-GlcA, R_2_=OH |
| **S43** | Astraisoolesaponin A_1_ |  | R_1_=Rha-(1→2)-Glc-(1→2)-GlcA, R_2_=OH |
| **S44** | Astroolesaponin F |  | − |
| **S45** | Astroolesaponin C_1_ |  | R=Glc-(1→2)-GlcA methyl ester |
| **S46** | Robinioside B |  | R=Rha-(1→2)-Glc-(1→2)-GlcA |
| **S47** | Astroolesaponin C_2_ |  | R=Rha-(1→2)-Glc-(1→2)-GlcA methyl ester |
| **S48** | Robinioside F |  | R=Rha-(1→2)-Glc-(1→2)-GlcA |
| **S49** | Astroolesaponin D |  | R=Rha-(1→2)-Glc-(1→2)-GlcA |
| **S50** | Astroolesaponin B |  | R=Rha-(1→2)-Glc-(1→2)-GlcA |
| **S51** | Astrolanosaponin B |  | − |
| **S52** | Astrolanosaponin E |  | − |
| **S53** | Lupeol |  | − |
| **S54** | Ursolic acid |  | − |
| **S55** | Soyasapogenol B |  | R= *β*-OH |
| **S56** | Soyasapogenol E |  | R= =O |
| **S57** | Huangqiyenin F |  | R= H |
| **S58** | Huangqiyenin G |  | R=OH |
| **S59** | Huangqiyenin E |  | R= *α*-OAc |
| **S60** | Huangqiyenin H |  | − |
| **S61** | Huangqiyenin J |  | − |
| **S62** | Huangqiyenin I |  | − |
| **S63** | Huangqiyenin K |  | − |
| **S64** | Huangqiyenin B |  | − |
| **S65** | Mongholicoside II |  | − |
| **S66** | 29-*O*-*α*-*L*-rhamnopyranosyl-abrisapogenol B |  | R_1_=H, R_2_=*β*-OH, R_3_=Me, R_4_=CH_2_O-*α*- *L*-Rha, R_5_=Me |
| **S67** | Astraolesaponin A |  | R_1_=*α*-L-Rha-(1→2)-*β*-D-Glc-(1→2)-*β*-*D*-ghucuronopyranosy1, R_2_=O, R_3_=Me, R_4_=Me, R_5_=Me |
| **S68** | Astraolesaponin B |  | R_1_=*α*-L-Rha-(1→2)-*β*-*D*-Glc-(1→2)-*β*-*D*-ghucuronopyranosy1, R_2_=*β*-OH, R_3_=Me, R_4_=Me, R_5_=CH_2_OH |
| **S69** | Astraolesaponin E_1_ |  | R_1_=*β*-*D*-Glc-(1→2)-*β*-*D*-ghucuronopyranosy1, R_2_=O, R_3_=CH_2_OH, R_4_=COOH, R_5_=Me |
| **S70** | Astraolesaponin E_2_ |  | R_1_=*β*-*D*-Xyl-(1→2)-*β*-*D*-ghucuronopyranosy1, R_2_=O, R_3_=CH_2_OH, R_4_=COOH, R_5_=Me |
| **S71** | Astraolesaponin D |  | R=Glc |
| **S72** | Astragaloside III |  | R=Glc-(1→2)-Xyl |

**References**

Aslanipour, B., Gülcemal, D., Nalbantsoy, A., Yusufoglu, H., & Bedir, E. (2017). Secondary metabolites from *Astragalus karjaginii* BORISS and the evaluation of their effects on cytokine release and hemolysis. *Fitoterapia*, *122*, 26–33.

Bi, Z. M., Yu, Q. T., Li, P., Lin, Y., & Gao, X. D. (2007). Flavonoids from the aerial parts of *Astragalus mongholicus*. *Chinese Journal of Natural Medicines*, *5*(4), 263–265.

Bian, Y. Y., & Li, P. (2008). Study on scavenging activities for superoxide anion radicalsand structure activity relationship of flavonoids from *Astragalus membranaceus* (Fish.) Bge.var. *mongholicus* (Bge.) Hsiao. *Chinese Pharmaceutical Journal*, *43*(4), 256.

Cao, Z. Z., Yu, J. H., Gan, L. X., & Chen, Y. Q. (1985). The structure of *astramembrannins*. *Acta Chimica Sinica*, *43*(6), 581–585.

Chen, G. M., Jiang, N., Zheng, J. P., Hu, H. M., Yang, H. B., Lin, A. Z., et al. (2023). Structural characterization and anti-inflammatory activity of polysaccharides from *Astragalus membranaceus*. *International Journal of Biological Macromolecules*, *241*, 124386.

Du, R. T., Xu, F., Wei, D. S., Wei, Y. X., Wang, Z.Y., & Wang, Z. B. (2024). Pharmacokinetics of two triterpenoid saponins and three flavonoids in *Astragalus membranaceus* leaves by UHPLC-MS/MS. *Journal of Pharmaceutical and Biomedical Analysis*, *251*, 116419.

Fu, J., Huang, L. F., Zhang, H. T., Yang, S. H., & Chen, S. L. (2013). Structural features of a polysaccharide from *Astragalus membranaceus* (Fisch.) Bge. var. *mongholicus* (Bge.) Hsiao. *Journal of Asian Natural Products Research*, *15*(6), 687-692.

Guo, K., He, X. F., Zhang, Y. P., Li, X. Z., Yan, Z. Q., Pan, L., et al*.* (2016). Flavonoids from aerial parts of *Astragalus hoantchy*. *Fitoterapia*, *114*, 34–39.

Hao, J., Li, J., Li, X. X., Liu, Y. X., Ruan, J. Y., Dong, Y. Z., et al*.* (2016). Aromatic constituents from the stems of *Astragalus membranaceus* (Fisch.) Bge. var. *mongholicus* (Bge.) Hsiao. *Molecules*, *21*(3), 354.

He, Z. Q., & Wang, B. Q. (1990). Isolantion and identifcation of chemical constituents of *Astragalus* root. *Acta Pharmaceutica Sinica*, *25*(09), 694–698.

Hirotani, M., Zhou, Y., Lui, H., & Furuya, T. (1994). Astragalosides from hairy root cultures of *Astragalus membranaceus*.100. studys on plant-tissue cultures. *Phytochemistry*, *36*(3), 665 –670.

Jiang, Y. P., Qi, X. H., Gao, K., Liu, W. J., Li, N., Cheng, N. B., et al*.* (2016). Relationship between molecular weight, monosaccharide composition and immunobiologic activity of *Astragalus* polysaccharides. *Glycoconjugate Journal*, *33*(5), 755–761.

Kim, A., Koo, J. H., Lee, J. M., Joo, M. S., Kim, T. H., Kim, H., et al*.* (2022). NRF2-mediated SIRT3 induction protects hepatocytes from ER stress-induced liver injury. *FASEB Journal*, *36*(3), e22170.

Kim, G. S., Kim, S. Y., Hong, Y., Lee, S. E., Lee, J. H., Lee, M. H., et al*.* (2013). Anti-Inflammatory *cycloartane*-type saponins of *Astragalus membranaceus* J Molecules. *Molecules*, *18*(4), 3725-3732.

Kim, J. S., Yean, M. H., Lee, E. J., Jung, H. S., Lee, J. Y., Kim, Y. J., et al*.* (2008). Two new *cycloartane* saponins from the roots of *Astragalus membranaceus*. *Chemical & Pharmaceutical Bulletin*, *56*(1), 105–108.

Kuang, H., Okada, Y., Yang, B., Tian, Z., & Okuyama, T. (2009). Secocycloartane triterpenoidal saponins from the leaves of *Astragalus membranaceus* BUNGE. *Helvetica Chimica Acta*, *92*(5), 950–958.

Kuang, H. X., Wang, Q. H., Yang, B. Y., Wang, Z. B., Okada, Y., & Okuyama, T. (2011). Huangqiyenins G-J, four new 9,10-secocycloartane (=9,19-Cyclo-9,10-secolanostane) triterpenoidal saponins from *Astragalus membranaceus* bunge leaves. *Helvetica Chimica Acta*, *94*(12), 2239–2247.

Lee, D., Lee, D., Choi, S., Lee, J. S., Jang, E. S., & Kang, K. S. (2019). Identification and isolation of active compounds from *Astragalus membranaceus* that improve insulin secretion by regulating pancreatic *β*-cell metabolism. *Biomolecules*, *9*(10), 618.

Lee, D. Y., Noh, H. J., Choi, J., Lee, K. H., Lee, M. H., Lee, J. H., et al*.* (2013). Anti-inflammatory cycloartane-type saponins of *Astragalus membranaceus*. *Molecules*, *18*(4), 3725–3732.

Lee, E. J., Yean, M. H., Jung, H. S., Kim, J. S., & Kang, S. S. (2008). Phytochemical studies on *Astragalus* root (2) : Flavonoids and a lignan. *Natural Product Sciences*, *14*(2)

Lee, J. A., Shin, M. R., Choi, J., Kim, M., Park, H. J., & Roh, S. S. (2023). Co-treatments of gardeniae fructus and silymarin ameliorates excessive oxidative stress-driven liver fibrosis by regulation of hepatic sirtuin1 activities using thioacetamide-induced mice model. *Antioxidants*, *12*(1), 97–97.

Li, R. F., Zhou, Y. Zh., Qiao, L. Fu., H. W., & Pei, Y. H. (2007). Chemical constituents of *Astragalus membranaceus* Bge. var. *mongholicus* (Bge.) Hsiao. *Journal of Shenyang Pharmaceutical University*, *24*(01), 20–22.

Li, Y. X., Li, Z. P., Yan, S. L., & Su, Y.F., (2017). Chemical constituents in roots of *Astragalus membranaceus*. *Chinese Traditional and Herbal Drugs*, *48*(13), 2601–2607.

Luo, Z., Su, M. Z., Yan, M., Shi, G. B., & Zhao, Q. C. (2012). Chemical constituents of *Astragalus membranaceus* var. mongholicus. *Chinese Traditional and Herbal Drugs*, *43*(3), 458–462.

Ma, X. F., Tian, X. M., Chen, Y. J., & Tu, P. F., (2005). Flavonoid constituents of *Astragalus membranaceus* var. *mongholicus*. *Chinese Traditional and Herbal Drugs*, *36*(09), 17–20.

Pei, Y., Li, R., Fu, H., Wang, J., & Zhou, Y. (2007). A new isoflavone glucoside from *Astragralus membranaceus* var. *mongholicus*. *Fitoterapia*, *78*(7–8), 602–604.

Sheng, Z. L., Liu, H., Li, B. L., Zhu, H., Jiang, Y. M., & Yang, B. (2024). Arabinogalacturonan in functional food “*Astragalus membranaceus* root”: Structure and immunomodulatory activity. *Food Hydrocolloids*, 159, 110725.

Song, C. Q., Zh, Z. R., Liu, D., & Hu, Z. B. (1997). Isoflavones from *Astragalus membranaceus*. *Acta Botanica Sinica*, *39*(8), 764–768.

Tian, H., Deng, Y. R., Zhou, K., & Cong, H. Y. (2016). Chemical constituents of *Astragalus membranaceus* var. *mongholicus*. *Chinese Journal of Experimental Traditional Medical Formulae*, *22*(7), 70–73.

Tu, T., Shen, J., & Jiang, J. (2009). Studies on constituents from the roots of *Astragalus membranaceus* (Fisch.) Bge. Hsiao. *West China Journal of Pharmaceutical Sciences*, *24*(5), 466–468.

Un, R., Horo, I., Masullo, M., Falco, A., Senol, S.G., Piacente, S., et al*.* (2016). Cycloartane and oleanane-type glycosides from *Astragalus* *pennatulus*. *Fitoterapia*, 109, 254–260.

Vasilev, H., Ross, S., Smejkal, K., Marsík, P., Jankovská, D., Havlík, J., et al*.* (2019). Flavonoid glycosides from endemic bulgarian *Astragalus aitosensis* (Ivanisch.). *Molecules*, *24*(7), 1419.

Wang, H. K., He, K., & Luo, Q. (1989). Studies on chemical composition of *Astragalus membranaceus* var. *mongholicus* (II). *Chinese Traditional and Herbal Drugs*, *20*(5), 6–8.

Wang, P. P., Wang, Z., Zhang, Z. P., Cao, H. Y., Kong, L. Y., Ma, W., et al*.* (2023). A review of the botany, phytochemistry, traditional uses, pharmacology, toxicology, and quality control of the *Astragalus memeranaceus*. *Frontiers in Pharmacology*, *14*, 1242318.

Wang, Q. H., Wang, X. L., Ao, W. L. J., Dai, N. Y. T., & Na, R. C. T. (2014). Chemical constituents of roots of *Astragalus membranaceus* (Fisch) Bge. var. *mongholicus*(Bge) Hsiao. *Chinese Pharmaceutical Journal*, *49*(5), 357–359.

Wang, Q. H., Han, N., Dai, N. Y. T., Wang, X. L., & Ao, W. L. J. (2014). Anti-inflammatory effects and structure elucidation of two new compounds from *Astragalus membranaceus* (Fisch) Bge. var. *mongholicus* (Bge) Hsiao. *Journal of Molecular Structure*, *1074*, 284–288.

Wang, Q. H., Han, N., Dai, N. Y. T., Wang, X. L., & Ao, W. L. J. (2014). The structural elucidation and antimicrobial activities of two isoflavane glycosides from *Astragalus membranaceus* (Fisch) Bge. var. *mongholicus* (Bge) Hsiao. *Journal of Molecular Structure*, *1076*, 535–538.

Wang, S. L., Peng, Y., Zhuang, Y. X., Wang, N., Jin, J. C., & Zhan, Z. J. (2023). Purification, structural analysis and cardio-protective activity of polysaccharides from *Radix Astragali*. *Molecules*, *28*(10), 4167.

Wang, T. T., Ruan, J. Y., Li, X. X., Chao, L. P., Shi, P. P., Han, L. F., et al*.* (2016). Bioactive cyclolanstane-type saponins from the stems of *Astragalus membranaceus* (Fisch.) Bge. var. *mongholicus* (Bge.) Hsiao. *Journal of Natural Medicines*, *70*(2), 198–206.

Wang, X., Tang, S. A., & Duan, H. Q. (2017). Studies on Astragalosides from *Astragalus membranaceus* (Fisch) Bge. *Journal of Tianjin Medical University*, *23*(6), 516–518.

Wang, Z. B., Zhu, W. B., Chen, Y. J., Yu, J. L., Ma, Z. P., Wu, G. S., et al. (2017). Flavonoids from the leaves of *Astragalus membranaceus*. *Chinese Traditional Patent Medicine*, *39*(8), 1634–1638.

Wang, Z. B., Zhai, Y. D., Ma, Z. P., Yang, C. J., Pan, R., Yu, J. L., et al*.* (2015). Triterpenoids and flavonoids from the leaves of *Astragalus membranaceus* and their inhibitory effects on nitric oxide production. *Chemistry & Biodiversity*, *12*(10), 1575–1584.

Wen, Y. H., Cheng, L., Zheng, D., Huang, X. Sh., & Han, L. (2010). Chemical constituents of *Astragalus membranaceus* var mongholicus. *Practical Pharmacy and Clinical Remedies*, *13*(2), 115–119.

Wu, J. C., Li, C. Y., Bai, L. S., Wu, J., Bo, R., Ye, M. Z., et al*.* (2021). Structural differences of polysaccharides from *Astragalus* before and after honey processing and their effects on colitis mice. *International Journal of Biological Macromolecules*, 182, 815–824.

Xiao, W. H., Han, L. J., & Shi, B. (2009). Isolation and purification of flavonoid glucosides from *Radix Astragali* by high-speed counter-current chromatography. *Journal of Chromatography B:Analytical Technologies in the Biomedical and Life Sciences*, *877*(8–9), 697–702.

Yu, Q. T., Li, P., Bi, Z. M., Luo, J., & Gao, X. D. (2007). Two new saponins from the aerial part of *Astragalus membranaceus* var . *mongholicus*. *Chinese Chemical Letters*, *18*(5), 554–556.

Zhang, L. J., Liu, H. K., Hsiao, P. C., Kuo, L. M. Y., Lee, I. J., Wu, T. S., et al*.* (2011). New isoflavonoid glycosides and related constituents from *Astragali Radix* (*Astragalus membranaceus*) and their inhibitory activity on nitric oxide production. *Journal of Agricultural and Food Chemistry*, *59*(4), 1131–1137.

Zhang, Y., Li, X., Ruan, J., Wang, T., Dong, Y., Hao, J., et al*.* (2016). Oleanane type saponins from the stems of *Astragalus membranaceus* (Fisch.) Bge. var. *mongholicus* (Bge.) Hsiao. *Fitoterapia*, *109*, 99–105.

Zhang, Y. Z., Xu, F., Liang, J., Tang, J. S., Shang, M. Y., Wang, X., et al. (2012). Isoflavonoids from roots of *Astragalus membranaceus* var. *mongholicus*. *China Journal of Chinese Materia Medica*, *37*(21), 3243–3248.

Zheng, S. S., & Wang, Z. T. (2011). Chemical constituents from the roots of *Astragalus membranace* (Fisch.) Bge. var. *mongholicus* (Bge.) Hisao. *Academic Journal of Shanghai University of Traditional Chinese Medicine*, *25*(5), 89–94.

Wang, Z. B., Zhai, Y. D., Ma, Z. P., Yang, Ch. J., & Pan, R. (2015). Triterpenoids and flavonoids from the leaves of *Astragalus membranaceus* and their inhibitory effects on nitric oxide production. *Chemistry & Biodiversity*, *12*(10), 1575–1584.

Zhou, Y., Liao, S. P., Zhang, Z. W., Wang, B., & Wan, L. H. (2016). Astragalus injection attenuates bleomycin-induced pulmonary fibrosis via down-regulating Jagged1/Notch1 in lungs. *Journal of Pharmacy and Pharmacology*, *68*(3), 389–396.

Zhu, Y. Z., Lu, S. H., Okada, Y., Takata, M., & Okuyama, T. (1992). Two new *cycloartane*-type glucosides, mongholicoside I and II, from the aerial part of *Astragalus mongholicus* bunge. *Chemical and Pharmaceutical Bulletin*, *40*(8), 2230–2232.
